# A Single-Neuron: Current Trends and Future Prospects

**DOI:** 10.3390/cells9061528

**Published:** 2020-06-23

**Authors:** Pallavi Gupta, Nandhini Balasubramaniam, Hwan-You Chang, Fan-Gang Tseng, Tuhin Subhra Santra

**Affiliations:** 1 Department of Engineering Design, Indian Institute of Technology Madras, Tamil Nadu 600036, India; pgupta1304@gmail.com (P.G.); balanandhinee@gmail.com (N.B.); 2 Department of Medical Science, National Tsing Hua University, Hsinchu 30013, Taiwan; hychang@mx.nthu.edu.tw; 3 Department of Engineering and System Science, National Tsing Hua University, Hsinchu 30013, Taiwan; fangang@ess.nthu.edu.tw

**Keywords:** single-neuron models, mapping, electrophysiological recording, isolation, therapy, micro/nanofluidic devices, microelectrode array, transfection, artificial intelligence

## Abstract

The brain is an intricate network with complex organizational principles facilitating a concerted communication between single-neurons, distinct neuron populations, and remote brain areas. The communication, technically referred to as connectivity, between single-neurons, is the center of many investigations aimed at elucidating pathophysiology, anatomical differences, and structural and functional features. In comparison with bulk analysis, single-neuron analysis can provide precise information about neurons or even sub-neuron level electrophysiology, anatomical differences, pathophysiology, structural and functional features, in addition to their communications with other neurons, and can promote essential information to understand the brain and its activity. This review highlights various single-neuron models and their behaviors, followed by different analysis methods. Again, to elucidate cellular dynamics in terms of electrophysiology at the single-neuron level, we emphasize in detail the role of single-neuron mapping and electrophysiological recording. We also elaborate on the recent development of single-neuron isolation, manipulation, and therapeutic progress using advanced micro/nanofluidic devices, as well as microinjection, electroporation, microelectrode array, optical transfection, optogenetic techniques. Further, the development in the field of artificial intelligence in relation to single-neurons is highlighted. The review concludes with between limitations and future prospects of single-neuron analyses.

## 1. Introduction

It would not be an exaggeration to state that the brain is the most complex structure present in the human body, with more than 100 billion neurons, ten times more glial cells, and hundreds of trillion nerve connections [1]. Neurons, the structural and functional unit of the nervous system, display a high complexity of cell diversity, and circuit organization rules. Rigorous research has demonstrated that the firing of even a single-neuron is sufficient to alter mammalian behavior or brain state [2]. Therefore, mapping individual neurons or sets of neurons with specifically distributed activity patterns displaying temporal precision is still an important and intriguing query. Single-neuron analyses in the mammalian brain requires crossing many technical barriers and involves four steps: (1) labeling individual neurons; (2) imaging at axon resolution levels in brain-wide volumes; (3) the reconstruction of functional areas or the entire brain via converting digital datasets of image stacks; (4) analysis to record morphological features of neurons with a proper spatial coordinate framework and also extract, measure, and categorize biological characteristics, i.e., neural connectivity. Neuron morphology becomes a native illustration of type of neuron, replicating their input-output connections. The great diversity, huge spatial span, and troublesome dissimilarities of mammalian neurons present several challenges in labelling, imaging and analysis [3]. 

A major challenge in studying single-neuron anatomy is that many pathological factors like stroke, trauma, inflammation, infection, and tumors have not been recognized or deliberated to be an effect of the individual neurons. General clinical studies have almost neglected the role of a single-neuron in the absence of relevant technology and tools to do so. Additionally, conventional in vitro and in vivo assays predominantly measured an average response from a population of cells. Such information may be informative in most studies but is not enough in cases where subpopulation information determines the behavior of the whole population [4].

In the last two decades, the rapid advancement of micro- and nano-technologies and their integration with chemical engineering, chemistry, life science, and biomedical engineering has enabled the emergence of a new discipline, namely the lab-on-a-chip or micro-total analysis system (μ-TAS). The lab-on-a-chip can not only manipulate cells precisely but also provide an environment for single-cell analysis with little sample and reagent consumption. Precise single-cell analyses, including cultivation, manipulation, isolation, lysis as well as single-cell mechanical, electrical, chemical and optical characterization, can be conducted with relative ease using micro/nanofluidic devices [5,6]. These single-cell analyses can help us to understand different biological contexts, such as the functional mutation and copy number effects of genes, and cell–cell or cell–environment interactions. All of these analyses are crucial for the development of cellular therapy and diagnostics [3,6,7]. Because stimulating just one neuron can affect learning, intelligence, and behavior, conventional assays that mainly analyze the average responses from a population of neurons in the brain may not be sufficient to provide the required information. Through single-neuron analyses, relationships across neuron modalities, holistic representation of the brain state, and integration of data sets produced across individuals and technologies can be achieved, and would greatly benefit future precision medicine development. Single-unit recordings from human subcortical or cortical regions contribute significantly in enhancing the understanding of basal ganglia function and Parkinson’s disease, neocortical function and epilepsy [8,9]. Single cell analysis was also employed in deciphering the neuronal signaling during epileptic form activity, owing to the alterations in metabolic state or level of arousal, and during normal cognition. After the first attempt at device implantation for single-cell recording in 2004, remarkable progress has been made. Based on the similar concept of single-cell recording and stimulation, the first intracortically directed two-dimensional (2D) cursor movements and simple robotic control were achieved by tetraplegia patients with an intracortical brain-computer interface. The studies conducted on patients with tremor medical condition using single-unit recordings helped in developing a better understanding of the role of individual basal ganglia and motor thalamic neurons, generating synchronized rhythmic firing in a tremor-associated manner [10].

The nervous system is composed of neurons and various supporting cells (oligodendrocytes, microglia, and astrocytes) of distinct morphology and neurochemical activity. Even a single sensory neuron activity can exactly predict the perceptions of animals [11]. Owing to the stochastic intercellular variation of the genome, epigenome, proteome and metabolome significantly cause variation in single-neuron response to therapeutics and the information is critical in precision medicine [12]. Therefore, isolation of distinct cells is a crucial step in single-neuron analyses, and the limitation factors associated with the process, such as efficiency or throughput, purity, and recovery, need to be improved.

This review article focuses on the latest developments in analytical technologies at the single-cell level in the nervous system. The technologies include modeling, isolation, mapping, electrophysiology, and drug/gene delivery (viral, optoporation, microinjection, and electroporation) at single-neuron levels. It also emphasizes therapeutic analysis and effect measurement using different micro/nanofluidic devices. Moreover, recent findings on the relationship between single-neurons and behavior and artificial intelligence will be summarized.

## 2. Single-Neuronal Models

Neuro-physiological research of single and multiple neurons has been carried out for centuries, yet the first mathematical model was established by Louis Lapicque in 1907 [13]. Based on the physical units of the interface, two categories of neuronal models were established. The electrical input–output membrane voltage model predicts the functional relationship between the input current and the output voltage. The other category, known as the natural or pharmacological input neuron model, relates the input stimulus (light, sound, pressure, electrical or chemical inputs) to the probability of a spike event. Even though many neuronal models were proposed, Hodgkin and Huxley’s model (the H&H model) of the neuronal membrane is considered the classic neural model for computational neuroscience to date.

The base of the Hodgkin–Huxley (H&H) model lies in Bernstein’s membrane theory, which was proposed in 1902 [14]. The H&H model established a relationship between the flow of ionic currents across the neuronal cell membrane and the voltage at the cell membrane. The major points from this theory were that the selective permeability of the cellular membrane allows only a particular concentration and type of ions to flow across the membrane. The voltage-current relationship was given by the formula:

(1) $$C_{m}\frac{\mathrm{dV}\left(t\right)}{\mathrm{dt}}=-\sum_{i}Ii_{\;}(t,V)$$

where C_m_ denotes membrane capacitance, I_i_ is the current through a given ion channel, t is the time and V stands for voltage.

The Hopfield model discussed the distributive memory mechanism and the output firing rate [15]. The FitzHugh–Nagumo model is a qualitative and simplified two-dimensional model of the H&H model, in which regenerative self-excitation of a single-neuron was described [16].

Hindmarsh’s and Rose’s model is characterized by periodical or chaotical bursts of spikes. This model is used to model other neuron processes, which can be either be autonomic or cognitive [17].

The above-mentioned models involve several complex nonlinear differential equations; furthermore, the required simulation time is considerably more significant than the information about the neural circuit behavior. On the contrary, few models exist where the neuron is considered only an element and ignoring the complicated morphology of the dendrites and ionic mechanism inside the neurons and all the synapses were simplified as inputs with different weights. It considered only the input–output relationship of neurons as the simplified model. These models can be divided into two parts: (1) artificial neuron: this does not elucidate the mechanism of living neural circuits, rather a constructed artificial neural networks with some specific function to solve a practical engineering problem; (2) realistic simplified neuron model: though it is not based on subcellular mechanisms, yet its main assumptions are realistic and based on the available knowledge about the behavior of living neurons. Realistic models are further categorized into two classes based on the method of coding. Temporal coding, i.e., Louis Lapicque’s integrate-and-fire model (1907) [18] and McCulloch and Pitts (1943) [19]; and neural coding considered as rate coding (output of a neuron is a continuous variable-firing rate of the frequency, for example, the Hopfield model (1994)) [16].

Briefly, the earliest model of a neuron, i.e., the Integrate and Fire model, represents neurons in terms of time. The firing frequency of a single-neuron was formulated as a function of constant input current, and it was given by frequency, f (I) = I/C_m_V_th_ + t_ref_ I, where C_m_ denotes the membrane capacitance, V_m_ the membrane potential, I the membrane current, and t_ref_ the refractory period.

The drawback of this model was that sometimes when it received a below-threshold signal, the voltage boost of the model was retained until another firing occurred (i.e., lack of time-dependent memory). Thus, another model, the Leaky Integrate and Fire model, was proposed by adding a leak term to the membrane potential to resolve the memory problem. Since the cell membrane is not a perfect insulator, a membrane resistance that forces the input current to exceed the threshold (I_th_ = V_th_/R_m_) cause the cell to fire. The firing frequency with the membrane resistance (R_m_) is given as

(2) $$f\;(I)=\left\{\begin{array}{c} 0\;I<I_{\mathrm{th}}\\ {\left[t_{\mathrm{ref}}-R_{m}C_{m}\log\left(1-\frac{v_{\mathrm{th}}}{R_{m}}\right)\right]}^{-1}\;I>I_{\mathrm{th}} \end{array}\;\right\}$$

where I_th_ and V_th_ denote threshold current and threshold membrane potential, respectively.

In summary, neurons can be considered as dynamic systems; therefore, nonlinear dynamical approaches are appropriate to justify the variation in their behaviors [20]. After going through all these models, the doubt becomes even more generic for deciding the basic unit of the nervous system: neurons or ion channels. Considering the variation in both neurons and ion channels, it would be justified to select either or some other entity as the basic unit of the nervous system in that particular or similar condition. Here, various single-neuron models and their categories and drawbacks are summarized in Table 1.

## 3. Behavior and Single-Neurons

It is accepted that behavior is the result of brain function and brain processes govern how we feel, act, learn, and remember [33]. The understanding of the performance and capacity of single cortical neurons on a perpetual task is a prerequisite for establishing the link between the brain and behavior [34,35]. Accumulating evidence in cortical research has shown that single-neurons match behavioral responses in discriminating sensory stimuli [36,37]. Cortical neurons show highly nonlinear responses as a result of probing by complex natural stimuli [38,39,40,41,42]. The first instance of stimuli-caused accurate discrimination was reported by Wang et al. using a songbird model to test the occurrence of natural behaviors involving complex natural stimuli [43,44]. In this context, the available sensory information in response to a song consists of a single spike train from all the neurons of the particular population. The quantification of all single spike trains helps in evaluating the contribution of single-neuron behaviors [45]. It can also be concluded that spike timing has a major impact on performance than spike rates and interspike intervals. Further temporal correlations in spike trains enhance the single-neuron performance in most cases [2].

Another study assessing the sensitivity in measurements of single middle temporal (MT) neurons towards the direction of discrimination suggests that a small number of neurons may account for a psychophysiological performance [46]. Nevertheless, sampling-based variation in the single MT neuron activity predicted a weak correlation with behaviors. The results suggest that the decision is dependent on the collective responses of several neurons [36]. Therefore Cohen et al. proposed two possible explanations for this paradox: (1) a long stimulation duration may overestimate neural sensitivity in comparison with psychophysical sensitivity; (2) mistaken assumptions due to insufficient data are possible when noise correlation level in MT neurons supports reverse directions. This quantitates the role of single-neurons in perception, dependent on the duration and the noise correlation [47]. Similarly, the variability of responses to visual stimuli in striate cortex neurons was analyzed, and the results showed that perceptual decisions on signals arise from a rather small number of neurons and are correlated across neurons [48]. The results also demonstrated the correlation between the pooled signals and neurons along with other neurons, and thus apparently the perceptual decision, generating high choice probabilities [49].

**Figure 1:**
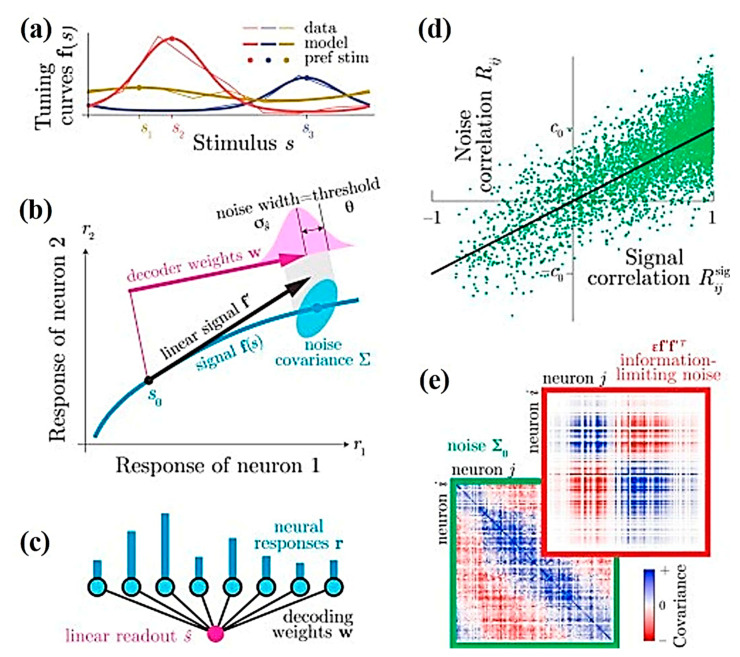
Model for neural responses and decoding: (**a**) tuning curves f(s) showing the mean neural responses to a stimulus s (thin lines), curve from the von Mises functions (thick curves) model with parameters including the preferred stimulus s_k_ (dots). (**b**) The relationship of two neurons generalizing to high-dimensional response spaces under varying stimulus s. (**c**) Linear decoding projects the neural responses, both noise and signal, towards a specific direction w for the estimation of ŝ of the stimulus. (**d**) The phenomenon of showing neurons having similar tuning has higher correlated fluctuations. Noise correlation coefficients R_ij_ between distinct neurons i and j are modeled as being proportional on average to the signal correlations Rijsig, with proportionality c_0_. (**e**) Two components to the noise covariance Σ: information-limiting correlations are distinguished; present along the signal direction f′ and therefore show covariance εf′f′^T^ (front, matrix boxed in red), and the remaining noise with covariance Σ_0_ (back, the matrix in the green box). The two types of noise show distinctive structures; apparent in the covariance matrices. The striations in the matrices correspond to the heterogeneous tuning curve amplitudes. Reprinted with permission from the authors of [50].

Similarly, Pitkow et al. predicted the role of single sensory neurons in behavior during discrimination tasks [50]. The notion is based on the limited sensory information from neural populations, either due to near-optimal decoding of a population with information-limiting correlations or by suboptimal decoding that is blind to correlations. Both possibilities involve different interpretations for the choice of correlations, i.e., the correlations between behavioral choices and neural responses. To assess this, experiments were conducted to record extracellular activities of single-neurons in the cerebellar nuclei (VN/CN), dorsal medial superior temporal (MSTd) and the ventral intraparietal (VIP) areas using epoxy-coated tungsten microelectrodes (FHC; 5–7 MΩ impedance for VN/CN, 1–2 MΩ for MSTd and VIP). The theoretical and experimental results shown in Figure 1 indicate the significance of noise correlations, which are governed by the response of the brain to these fundamental changes followed by processing sensory information [50].

Single-neuron studies also illustrated the role of interval-selective neuron population for revealing changes in behavioral significance temporal patterns of presynaptic input. The behavioral sensitivity within the millisecond timescale in natural scallops was observed at the minimum, because of the midbrain neuron population acting as temporal filters intended for electrical communication signals. Variation of an order of interpulse intervals (IPIs) and the addition of even 1 ms jitter to natural scallops have the scope to affect both behavior and single-neuron responses even by different individuals. An amount of poorly decodable information is encoded in sensory and motor circuits via temporal patterns of spikes [51].

In most of the models, the precise control of the temporal input pattern onto temporally selective neurons in vivo is tough. Therefore, this limitation was overcome by the mormyrid electric fish model, where similar temporal patterns were found for presynaptic inputs against interval-selective central neurons and electrosensory stimuli [52]. Furthermore, based on single-neuron analysis, electric communication signals are tunable according to behavioral relevance. This shows that temporal patterns of presynaptic input onto interval-selective neurons can be tuned along with recording the responses of these neurons to input patterns, present while natural communication behavior. The results also show coherence between earlier findings of auditory and electrosensory pathways related to discriminating among scallops from different individuals. The neuron spikes of songbird field L neurons, grasshopper auditory receptors and higher-order neurons, and wave-type electric gymnotiform fish (which evolved their electric sense independently of mormyrids) hindbrain neurons would help in identifying conspecific signals by each individual. However, it is beyond the scope of single-neuron variation, reacting to natural signal differences to measure the power of single-neurons corresponding to specific temporal alterations [53].

## 4. Single-Neuron Isolation

Depending on the application, several techniques have been employed to isolate single-neurons. The pipette approach is the most commonly exploited single-neuron isolation method. Pipetting is a flexible approach that allows applications such as the functional electrophysiology, imaging, and transcriptomics of neurons to be achieved simultaneously [54]. The pipette isolation process is well equipped with video recording and image documentation facility and is thus suitable for post-capture quality control. Moreover, the protocol can be adjusted for isolating subcellular structures, such as dendrites and even biomolecules. Isolated ribonucleic acid (RNA) samples from single-neurons, allows the generation of transcriptomics data using either microarray or RNA sequencing (RNA-seq) techniques. Single-neuron transcriptomic analyses provide deep insight views into cell function and enable sorting out the global variations among single-neurons. The isolation of RNA from single cells in intact tissue and the subsequent handling of a large number of RNA samples require advanced instrumentation. Protocols, including the collection softwares, photoactivated localization microscopy (PALM) and laser capture microdissection techniques, have been developed for isolating single-neurons from cultures and tissue slices via pipette capture [55]. Laser capture microdissection is an indirect touch technique to isolate a single cell without altering or damaging the native morphology and chemistry of the sample as well as surrounding cells; this therefore makes this technique suitable for isolating cells for downstream processing, i.e., DNA genotyping and loss of heterozygosity (LOH) analysis, RNA transcript profiling, cDNA library generation, proteomics discovery and signal-pathway profiling [56]. The method employs a focused laser beam to melt the thin transparent thermoplastic film placed on a cap on the target cells. The melted film infuses with the underlying selected cell and allows the transfer of the attached targeted cells to a microcentrifuge tube for further downstream processing. Individual dopaminergic neurons or the ventral tegmental area are successfully isolated by the blend of infrared capture laser and the ultraviolet cutting laser exposure on polyethylene naphthalene membrane slides [56]. The support membrane maintains the integrity of the desired region while lifting during the sample collection.

Another approach to isolate single-neurons uses dielectrophoresis (DEP)-based microfluidic devices. Dielectrophoresis is an electro-kinetic phenomenon based on movement (trapping, alignment, and patterning) of polarizable particles (in this case, cells) under the influence of a non-uniform electric field. The technique employs minimal electric field intensity and therefore does not cause damage to neurons. Nevertheless, the low ionic strength buffer used in DEP may sometimes result in high susceptibility of the neurons towards the physiochemical environment (i.e., pH, temperature, humidity, and osmotic pressure) as well as transfection and transduction outcomes. Additionally, observation of morphology and activity of cultured neurons in DEP experiments under an inverted microscope may be limited due to non-transparent electrodes and substrate used in the devices [57]. The problem was overcome, however, using a fully transparent DEP device fabricated with indium tin oxide (ITO) multi-electrode arrays and polydimethylsiloxane (PDMS). Such a device can be mounted on a microscope equipped with an incubator system to avoid contamination. The DEP electrode array traps and releases neurons (one at a time/electrode), as shown in Figure 20. The segregated single-neurons can be cultured and monitored over time, allowing the screening of various electrophysiological parameters and enabling detailed neurological studies [58].

## 5. Single-Neuron Mapping

The complex architecture of the human brain and how the billions of nerve cells communicate have perplexed great minds for centuries. However, in recent years, the rapid development of many new technologies is allowing neuroscientists map the brain’s connections in ever-available detail. Brain navigation has become more accessible than ever and we are now able to fly through significant pathways in the brain, perform comparison among circuits, scale-up the exploration of cells comprising the region, and the functions depending on them. The Human Connectome Project (HCP), targets creating a complete neuron map involving structural and functional connections in vivo, within and across individuals, providing an unparalleled compilation of the neural data.

From each synapse to single-neurons to long-range neural networks, combining individual maps could create a “meta-map” that provides something closer to a full, detailed computer simulation of brain networks. The use of high-end brain mapping technology CLARITY, in addition to light microscopy, has allowed researchers to draw limited maps for specific neurons of interest, even in large brains [59]. The CLARITY is a technology to transform intact biological tissue into a hybrid form where tissue component removal and replacement takes place with exogenous elements for better accessibility and functionality. The light microscope is not competent to decipher all at the nanometer scale—thin wires and synapses, connecting neurons—only electron microscopy (EM) possess the power to do that. “The wires define the computations that are possible by the circuits”, says Albert Cardona, a group leader at the Howard Hughes Medical Institute’s Janelia Research Campus. The subjects studied in connectome research range from living individuals to the preserved brains of tiny animals such as worms and flies. The investigative technologies are also diverse, ranging from light and electron microscopy to Magnetic Resonance Imaging. Regardless of the approaches, painstaking efforts have to be exerted to build an atlas, even with the aid of powerful computation tools. Although the roles of single-neurons in brain functioning have not been fully elucidated, a high-resolution neural connectome map that precludes redundancy to facilitate clear messaging is essential to understand the brain. At first, charting and understanding the full wiring diagram of the brain seems to be an impossible task, yet recent technological advancements make it optimistic without requiring decades to complete. Such an ambition also prompts efforts to overcome major challenges in robustness and reproducibility during sample preparation, handling, and analysis. Technologies concerning automatic image data acquisition and efficient data storage and analysis tools also need to be developed. This section will briefly discuss these challenges and possible solutions, together with novel imaging techniques to meet the challenge of single-neuron mapping in the nervous system [60].

**Figure 2:**
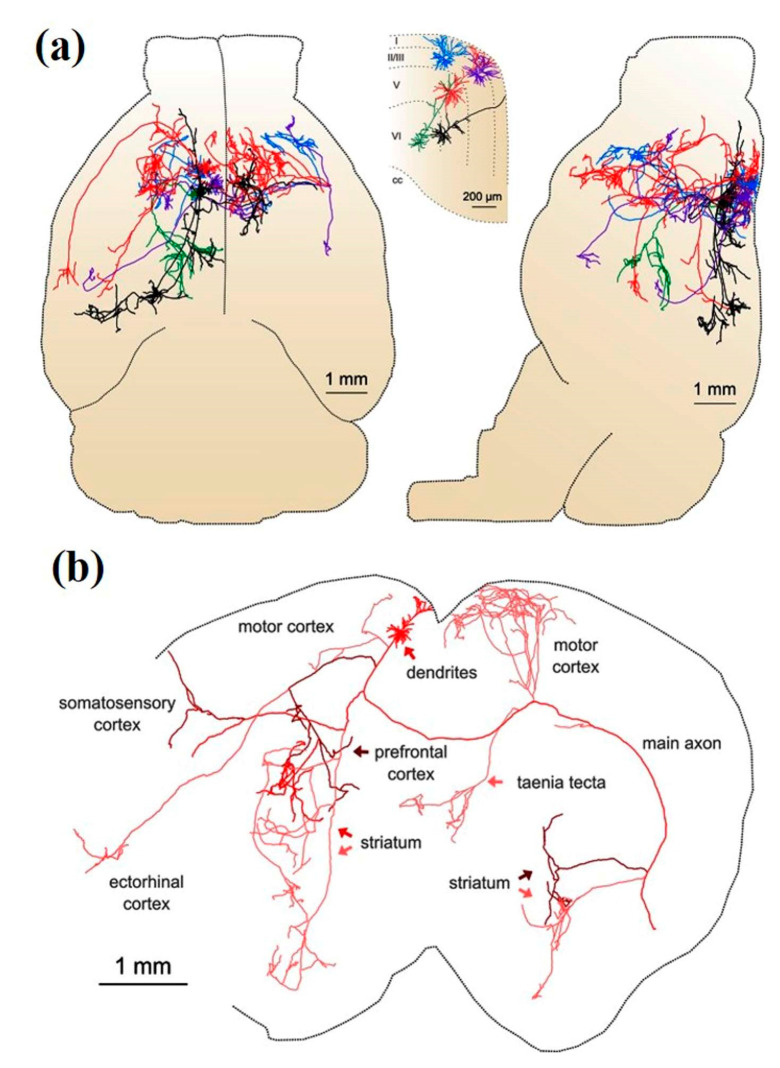
Complete reconstruction of axonal morphology. (**a**) Complete reconstruction of the five projection neurons, superimposed on a horizontal (left) and sagittal (right) position while imaging the mouse brain. The subset comprises pyramidal neurons in layer II (blue, purple), layer V (red, black), and layer VI (green). (**b**) Axonal and dendritic reconstruction of the layer, five pyramidal cells (colored red in (**a**) presented in the coronal plane. The black dashed line depicts the profile of the coronal section at the rostrocaudal position of the cell body. Colored segments highlight axonal arbors initiating from common branch points. Reprinted with the permission of the authors of [63].

Kebschull et al. highlighted the importance of understanding the fundamental neural wiring network to figure out how the brain works [61]. Similarly, Professor Toga pointed out that brain mapping is similar to traditional cartography that shows even the footpaths and steppingstones of individual neurons and synapses at resolutions of a few nanometers [62]. Neuronal cell types are the nodes of the neural circuit regulating the information flow through long-range axonal projections in the brain. Single-cell and sparse-labeling techniques have been employed to reconstruct long-range individual axonal projections in various parts of brain, i.e., the basal ganglia, neocortex, hippocampus, olfactory cortex, thalamus, and neuromodulatory systems, with limited reliability and throughput of axonal reconstruction due to labeling restrictions executed on one or very few neurons within a single brain. The manual tracking of individual distinct segments among consecutive slices generally gets deformed or damaged during standard histological processing techniques. Although the reliable and efficient reconstruction of long-range axonal projection can be achieved by visualizing neurons in continuous whole-brain image volumes. The serial two-photon (STP) tomography-based fast volumetric microscopy provides high-resolution imaging in complete three-dimensional space in a large volume of tissue, thus minute axonal collaterals may be unambiguously tracked to their targets [63]. Along with using this technique, high intensity sparse neuronal labeling, the new tissue clearing method, and bioinformatics tools to process, handle, and visualize huge imaging data lead to a suitable platform to efficiently reconstruct the axonal morphology. This was demonstrated by reconstructing the extensive, brain-wide axonal arborizations of diverse projection neurons present in the motor cortex within a mouse brain, as shown in Figure 2 [63].

**Figure 3:**
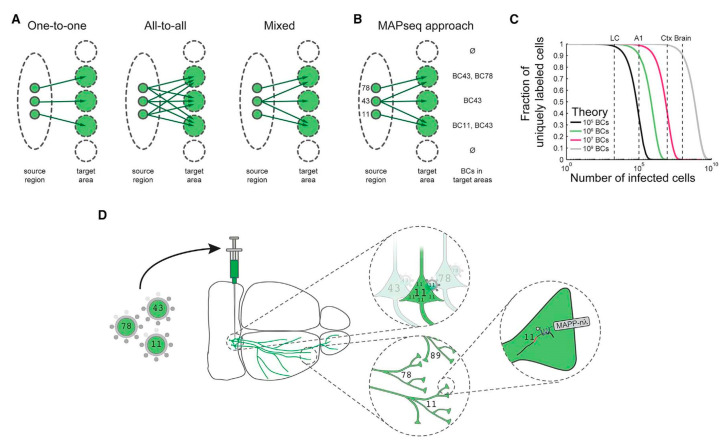
Multiplexed Analysis of Projections by Sequencing (MAPseq) procedure for mapping single-neuron projections. (**A**) Various underlying projection patterns develop identical bulk mapping. (**B**) Random labeling of single neurons with barcodes. (**C**) The expected fraction of uniquely labeled cells is given by F = (1-1/N)(k-1), where N is the number of barcodes and k is the number of infected cells, assuming a uniform distribution of barcodes. (A1, primary auditory cortex; Ctx, neocortex). (**D**) In MAPseq, neurons are infected at low MOI with a barcoded virus library. Barcode mRNA is expressed, trafficked, and can be extracted from distal sites as a measure of single-neuron projections. Reprinted with the permission from [61].

Manual intervention of the dataset remains a major bottleneck for neuronal reconstruction. A specialized custom reconstruction software generally takes 1–3 weeks to reconstruct a complete complex cortical neuron from precisely stitched brain volumes [3]. To increase the throughput of single-neuron mapping, an RNA sequencing-based method was developed [61]. Zador et al. implemented a Multiplexed Analysis of Projections by Sequencing (MAPseq) method, based on speed and the parallelization of high-throughput sequencing for brain mapping [64]. Multiplexing can be achieved in MAPseq by short, random RNA barcodes for unique and distinct labeling of individual neurons [64,65,66]. Barcodes are important as their diversity grows in an exponential manner as per the sequence length, overpowering the restricted resolvable color range. For example, the 30 nt sequence has a potential diversity of generating 4^30^–10^18^ unique barcode identifiers, way more than what is needed to distinguish 10^8^ neurons in a mouse brain [67]. As fast and inexpensive high-throughput sequencing can differentiate the barcodes, the MAPseq has the potential to identify the projections of millions of individual neurons in a brain simultaneously. In MAPseq, neurons are uniquely labeled by injecting a viral library encoding an assorted group of barcode sequences in a source region (see Figure 3). The highly expressed barcode mRNA is transferred to the axon terminals located at distal target projection regions. Later, the barcode mRNA is extracted from the injection site or target area and sequenced to read out the single-neuron projection pattern, as shown in Figure 3. The target should be precisely dissected to achieve higher spatial resolution. Like green fluorescence protein (GFP) tracing, MAPseq is unable to trace fibers of passage, therefore leaving out large fiber bundles while dissecting the target areas is critical in the study. This method takes less than a week to determine the brain-wide map of projections of a particular area, allowing efficient single-neuron circuit tracing [61].

As described earlier, along with the limitation of spatial resolution due to micro-dissection, MAPseq might show inherent sensitivity. Therefore, neuronal reconstructions based on microscopy ensure the gold standard for deciphering connections as well as the spatial organization of axonal projections. Optical imaging approaches, in combination with genetic tools and computational techniques, are starting to enable such global interrogations of the nervous system [68]. Haslehurst et al. employed a custom-built light fast sheet microscope (LFSM) using synchronized galvo-mirror and electrically tunable lens. The high-speed image acquisition facilitated the dendritic arborization of a living pyramidal neuron for 10 s in mammalian brain tissue at configurable depth. Post-hoc analysis represented localized, rapid Ca^2+^ influx events occurring at various locations and their spread or otherwise through the dendritic arbor [69]. Prior to this, Ahrens et al. used high-speed light-sheet microscopy for image the neurons in intact brain of larval zebrafish with single-neuron resolution. They could image as many as 80% of neurons at single cell resolution, while the brain activity was being recorded once every 1.3 s by genetically encoded calcium indicator GCaMP5G. The indicator is expressed under the influence of the pan-neuronal elavl3 promoter. The SiMView light-sheet microscopy framework plays a key role in volumetric imaging during this fast, three-dimensional recording from an entire larval zebrafish brain, mostly consisting of ~100,000 neurons [70]. The chemically cleared fixed brain tissues were also imaged with single-cell resolution using light sheet microscopy and the reconstructions of dendritic trees and spines in populations of CA1 neurons in isolated mouse hippocampi was performed [71].

Multiple variants of super-resolution microscopy, including structured illumination microscopy (SIM), stimulated emission depletion microscopy (STED), and photoactivated localization microscopy (PALM)/stochastic optical reconstruction microscopy (STORM), each with special features, have overcome the drawbacks of conventional microscopy and have helped remarkably in neuroscience to decipher mechanisms of endocytosis in nerve growth and fusion pore dynamics, and also describe quantitative new properties of excitatory and inhibitory synapses [72,73]. Though most recently, a super-resolution microscopy approach was developed to unravel the nanostructure of tripartite synapses with direct STORM (dSTORM) using conventional fluorophore-labeled antibodies. As a result, the reconstruction of the nanoscale localization of individual astrocytic-glutamate transporter (GLT-1) molecules surrounding presynaptic (bassoon) and postsynaptic (Homer1) protein localizations in fixed mouse brain sections was achieved [74].

**Figure 4:**
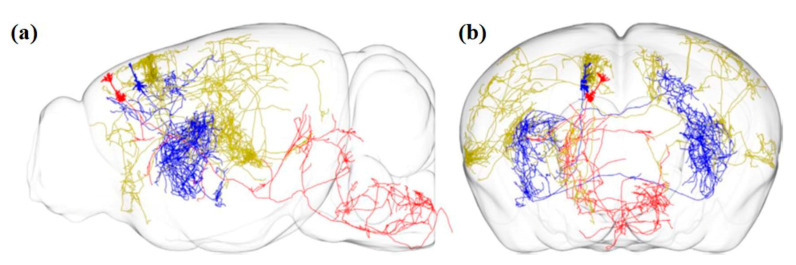
Axonal arbor for three cortical projection neurons of layer five of the motor cortex collapsed in the sagittal plane (**a**) and coronal plane (**b**). Intratelencephalic neurons shown in yellow and green color are projected to other cortical areas and the striatum with a higher level of projection heterogeneity. Pyramidal tract neurons (red) are connecting the motor cortex with hindbrain and midbrain. Reconstructions are retrieved from MouseLight Neuron Browser [76]. Total axonal lengths of shown neurons are 44.7, 30.1 and 13.4 cm for yellow (ID: AA0100), blue (ID: AA0267), and red (ID: AA0180), respectively. Reprinted with the permission of [75].

Economo et al. imaged the whole brain with a sub-micrometer resolution with the help of serial two-photon tomography. The sensitivity of the method also allowed manual tracing of fine-scale axonal processes through the entire brain, as shown in Figure 4 [63,75].

**Figure 5:**
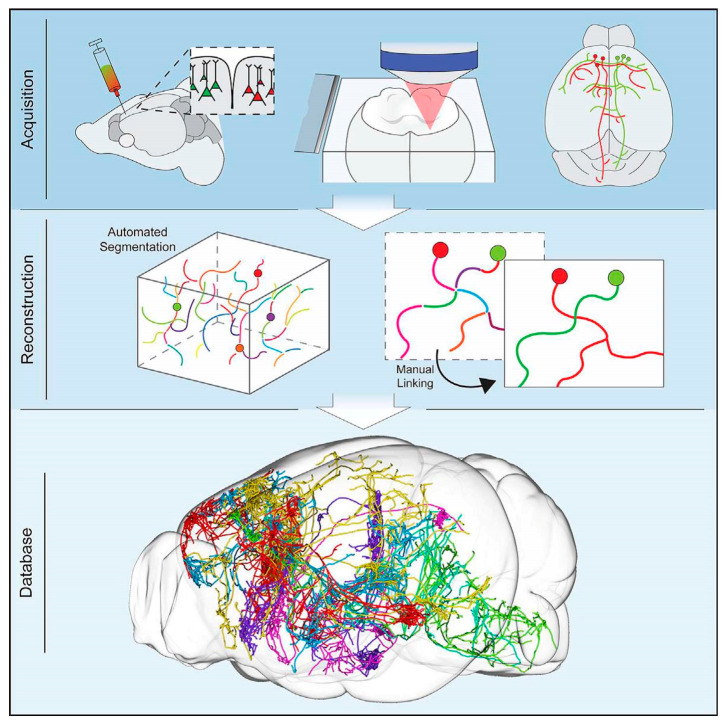
Schematic representation of 1000 projection neurons reconstruction deciphering new cell types and long-range connectivity organization present in mouse brain. Reprinted with the permission of [77].

Further improvement has been made to develop a semi-automated, high-throughput reconstruction method to reconstruct >1000 neurons in the neocortex, hippocampus, hypothalamus, and thalamus. Figure 5 shows the schematic representation of reconstruction for 1000 projection neurons. The reconstructions are made available in an online database MouseLight Neuron Browser with a wide visualization and inquiry window [76]. The findings discovered new types of cells and established innovative organizational doctrines which handle the connections among brain regions [77].

## 6. Electrophysiological Recording

**Figure 6:**
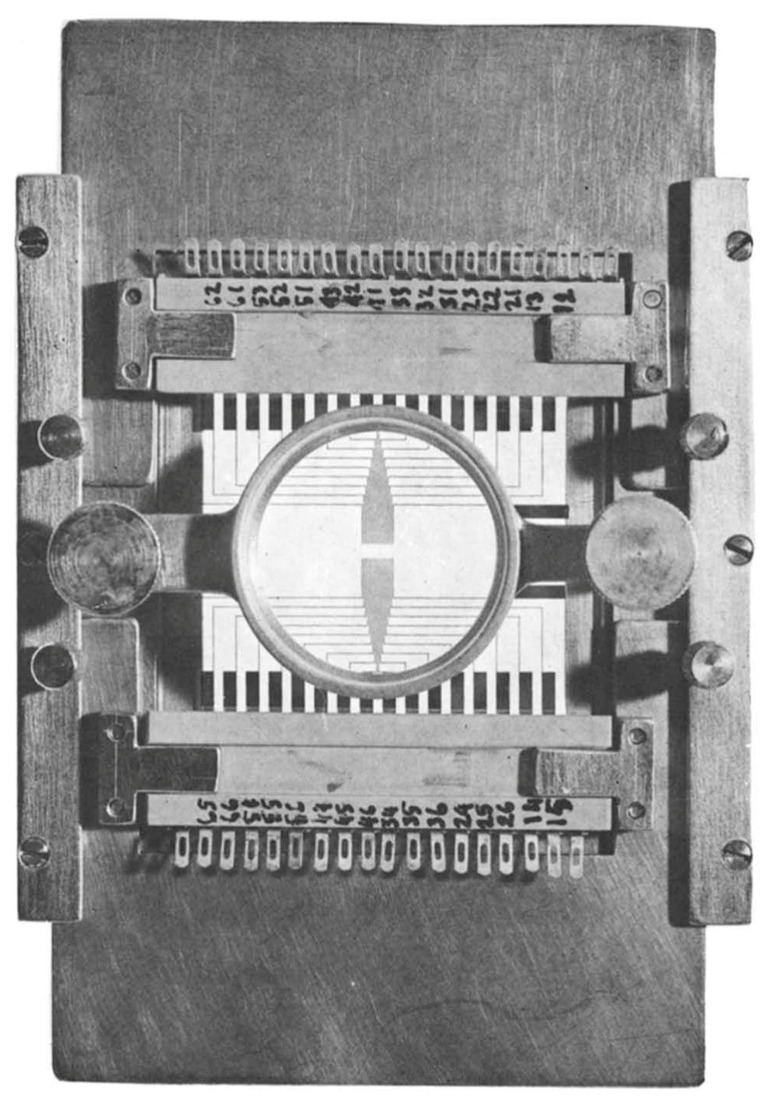
The microelectrode array consists of 36 photoetched microelectrodes with electrode holders, culture ring, and contact strips to study the single-unit neuronal activity. Reprinted with the permission of [81].

The electrical nature of neurophysiology was first identified by Italian scientist Luigi Galvani in 1794 [78]. The first recording of extracellular action potentials was carried out using a tungsten electrode of sub microns diameter tip sizes by Hubel [79]. The study of individual neurons provides high spatiotemporally resolved activities, which help us to study the inner working function of the brain [80]. In 1977, Gross et al. designed a two-dimensional multi-microelectrode system to study the single-unit neuronal activity. The microelectrode system, as shown in Figure 6, and it was fabricated by a photoetching process followed by galvanic plating of gold to produce a high-density gold electrode array. The 12 µm wide and 2 µm thick gold conductor de-insulated at the tip with a single laser shot. The de-insulated conductor had an impedance at 1 kHz of approximately 4 MΩ for a smooth gold surface and 2 MΩ for a rough gold surface facilitating electrophysiological recordings from more than 30 neurons [81].

Traditionally, a technique called stereotrode was designed in 1983 to record the extracellular action potentials of the nervous system—the ratio of the distance between the cells and two electrode tips governs the spike-amplitude ratios—while recording via both the channels. For this study, the electrode pair fabricated from Teflon-insulated platinum-iridium wires of 25 µm diameter, with an impedance of 1 MΩ at 1 kHz was used. The recordings provided a study on the statistical interaction among the spike trains of a local set of neurons, which improves the quality of the chronic unit recordings [82]. In 1999, a neurochip with a 4 × 4 array of metal electrodes recorded and stimulated electrical activity in individual neurons with no crosstalk between channels. By using this device, the action potentials recorded from individual neurons were detected with a signal-to-noise ratio of 35–70:1. But the chip showed the survival of neuron rarely beyond 7 days [57].

Considering the scope and limitations of this review paper, the electrophysiological recordings from single-neuron level are categorized into two parts: in vitro recording and in vivo recording. The in vivo part also includes single-neuron recordings from brain slices and ex vivo.

### 6.1. In Vitro Recording

**Figure 7:**
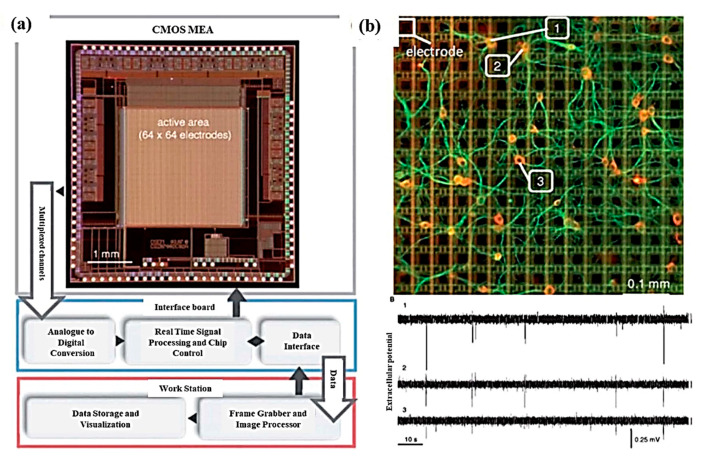
(**a**) Electrophysiological platform integrated with a complementary metal–oxide–semiconductor (CMOS) microelectrode array chip, the interface board, and a workstation. (**b**) Immunofluorescence imaging of single-neurons on the chip and the electrophysiological activity of three selected neurons. Reproduced from [83] with the permission of the Royal Society of Chemistry.

With the advancement of technology, multielectrode platforms have been developed with thousands of electrodes for the stimulation and recording of cell activity. In vitro single-neuron recording can be carried out using a 64 × 64 microelectrode array consisting of a total of 4096 microelectrodes with high spatial (21 µm of electrode gap) and temporal resolution (0.13 ms to 8 µs for microelectrodes of 4096 and 64 respectively), as depicted in Figure 7a,b. With high neuronal populations, the possibility to study an individual neuron is difficult; hence, low neuronal culture populations are preferred for single unit activity study. Also, single-pixel electrodes were selected to record signals from single-neurons and were interpreted to identify spiking and bursting events [83]. Mitz et al. conducted experiments on the frontal pole cortex of macaque monkeys to record the single-unit activity and neurophysiology of single cells. The recordings were performed by inserting 4–13 moveable microelectrodes, and their position was confirmed by magnetic resonance imaging. The monkeys experiments were conducted to perform three tasks out of which two were strategy tasks, and one was the control task, and the activity of isolated neurons was recorded [84]. Similarly, microelectrode arrays with 59,760 platinum microelectrodes [85], a complementary metal–oxide–semiconductor (CMOS) multielectrode array (MEA) chip with 16,384 titanium nitride electrodes [86], and 26,400 bidirectional platinum electrodes [87] also exist for in vitro electrophysiological recording with single-neuron resolution. The results depicted the activation of single-neuron arrays via intracellular stimulations. Electrophysiological recording shown the potential of tracing spiking neurons within neuronal populations, which is helpful to reveal the connection and activation modalities of neural networks [88]. Further, for better electrical interfacing with the aim of minimizing neuronal membrane deformation during the intracellular access, a vertical nanowire multi electrode array (VNMEA) was developed. This platform is capable of neuronal activation with the spatially/temporally confined effect along with recording its activity [89]. Next-generation non-invasive electrophysiology recording platforms are developed in the form of a thin-film, 3D flexible polyimide-based microelectrode array (3DMEA), facilitating the formation of 3D neuron networks. The array consists of 256 recording or stimulation channels. The action potential spike and burst activity were recorded for human-induced pluripotent stem cell (hiPSC)-derived neurons and astrocytes entrapped in a collagen-based hydrogel and seeded onto the 3DMEA, over 45 days in vitro [90].

### 6.2. In Vivo Recording

Further, to study in vivo single-unit activity, stereoelectroencephalography probes with a parallel batch of polyimide-platinum cylindrical microelectrodes of 800 µm diameter were used. The configurations of up to 128 electrode sites were set up to study the single-neuronal activity when various tasks were performed [91]. Similarly, the stereoelectroencephalography probes with 18 platinum microelectrodes of 35 µm diameter with an impedance of about 255 kΩ at 1 kHz were designed to measure the single-neuron activity to study focal epilepsy [92]. The dendritic integration of neurons can be studied only if the inhibitory and excitatory synaptic inputs of individual neurons are measured. For this measurement, an extracellular high-density microelectrode array of 11,000 electrodes were fabricated with firing at microsecond resolution. The presynaptic potentials were measured for a patched single-neuron with high reliability by eight randomly selected electrodes from the array [93]. In a study, the electrophysiological recordings of single-neurons were carried out by the patch-clamp technique followed by RNA sequencing to reveal the physiological and morphological properties of an individual neuron [94]. Single-neurons that were electrically transfected with plasmid DNA using micropipettes were studied for electrophysiological recordings. The membrane potential of the transfected and non-transfected neurons was examined to check whether there was any discrepancy, and was found to be −72 mV and −71 mV, respectively. Also, the electrophysiological properties of transfected and non-transfected neurons in brain slices were recorded and it was noted that the electroporation process did not affect the characteristics of the individual neurons [95].

Multielectrode array can record the two-dimensional range of action potential propagation in single-neurons via averaging the signals recorded extracellularly, which were detected by multiple electrodes. Here, medium-density arrays with an electrode pitch of 100 ± 200 µm were used to detect action potentials from single-axonal arbors. This non-invasive extracellular recording helped to identify the spiking of an individual neuron and it can be used to observe variations because of degeneration and in disease-models [96]. Electrophysiological recordings of single-neurons in the cortical and subcortical of mammalian animals were conducted using various conformations of microelectrode matrices. Microelectrodes were made from Teflon coated stainless steel with 50 µm diameter with two parallel rows of eight microwires each. They were inserted as chronic implants in rat primary (SI) somatosensory neurons to perform recording in the ventral posterior medial nucleus of the thalamus and sub-nuclei of the trigeminal brain stem complex with a configuration consisting of eight or 16 microwires. The advantage of this neuro technique is that the neural recordings may help to reconstruct neural engrams [97].

**Figure 8:**
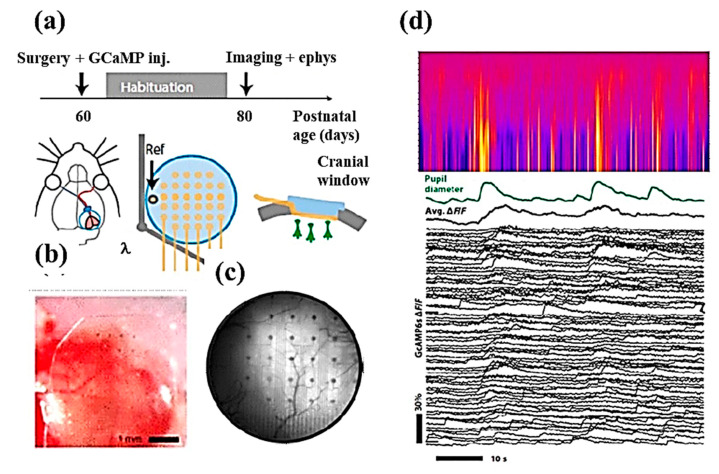
(**a**) Position of multielectrode array (MEA) on the mouse brain and the cranial window. (**b**) Implantation of the MEA in the mouse brain. (**c**) Epifluorescence of the brain and the surrounding areas. (**d**) Simultaneous electrophysiological recording, arousal, and two-photon imaging with single-neuron Ca^++^ activity. Reprinted with the permission of [98].

Qiang et al. developed a transparent microelectrode array to simultaneously record electrophysiological study as well as imaging by using the two-photon technique, as shown in Figure 8. The transparent microelectrodes were made from the Au nanosphere, and polyethylene oxide (PEO) was used for close packing of nanospheres. A 32-channel microelectrode array with 80 µm in diameter and an impedance of 12.1 kΩ was used with high spatial distribution and resulted in high uniformity neural recordings. This transparent microelectrode arrays provided high temporal and spatial resolution with high sensitivity and selectivity for recording single-neuronal signals, as shown in Figure 8d [98]. To measure the single-neuron membrane potential, simultaneous multi patch-clamp and multielectrode array recordings were combined. This system consisted of a 60-electrode array with 30 µm electrode diameter and a pitch of 0.5 mm. The multielectrode array provides spontaneous firing activity to the neurons, and the system can record simultaneously extracellular and intracellular activities of the patched neuron [99].

Direct interfacing with the nervous system may facilitate the extraction of millions of millisecond-scale information from single-neurons that will greatly benefit the personal diagnosis and follow-up treatment. Even though modern techniques have been developed to achieve good spatial resolution, such as structural and functional MRI, and temporal resolution, such as electroencephalography and magnetoencephalography, the measurement of the action potential and firing pattern in single-neurons have not been completely resolved. Hence, numerous animal models are still being used in the study to understand the physiological activities of small populations of individual neurons. In 1971, the first single-unit activity recording in epilepsy patients was performed by inserting an electrode with fine wire through the center of the brain. This study found that when the seizures were approaching the neuronal action potentials were periodic with the frequency associated with the time and phase of the gross waves. This can be related to the changes in the interaction between groups of neurons in neuronal networks [100]. After two decades, Fried et al. in 1999 described a technique that measured extracellular neurochemicals by cerebral microdialysis along with simultaneous measurement of electroencephalographic recordings and single-unit activity of neurons in the selected target. They conducted this study in 42 patients with a total of 423 electrodes, and the number of electrodes for each person varied from six to 14. These electrodes for single-unit neuron activity recording have four to nine 40-µm microwires that were made of a platinum alloy. The tests were conducted at 5–10 min intervals during seizures, cognitive tasks, sleep-waking cycles, and the release of amino acids and neurotransmitters for the evaluation of patients with a head injury, epilepsy, and subarachnoid hemorrhage [101]. Another single-neuronal recording platform, known as the Utah array, consisted of etched silicon array of 100 probes and was developed to record the patterns from individual neurons. A Utah array with 96 microelectrode contacts has been placed in the center of the brain to record the neuronal activity and hence monitor the symptoms of Parkinson’s disease [102,103]. The Utah array has also been implanted intracortical, directed by two-dimensional cursor movements to record the single-unit activity in epilepsy patients. In these studies, the Utah arrays recorded signals from different single units rather than from different layers of the brain. One interesting finding obtained from this epilepsy study was that there was an interplay between multiple classes and types of neurons, but the seizures did not propagate to the outside regions [104,105].

Furthermore, a relationship between single-neuron spiking and interictal discharges was established by analyzing the spiking rates of neurons that were recorded between seizures and during the seizures. A total of 90 neurons were recorded extracellularly from 17 awake patients, and it was noted that few neurons showed increased spiking rates during epileptic activity [8]. The drug-resistant focal epilepsy can be treated with stereoelectroencephalography probes by studying the single-unit activity recorded during epileptic seizures. The trials were conducted on a monkey by inserting three polyimide platinum cylindrical probes with varying electrodes sites [32,64] and the recordings were made. The single-unit activity of the neurons measured from the device was used to improve the precision of epileptic focus detection [91,92]. Various experiments were conducted on 36 patients with advanced Parkinson’s disease, who underwent microelectrode-guided posteroventral pallidotomy. The microelectrodes were placed to measure the single-unit recording and this was analyzed under various firing patterns, frequencies, and the response of movement-related activity. Magnetic resonance imaging was carried out to examine the size and location of the lesions [106].

**Figure 9:**
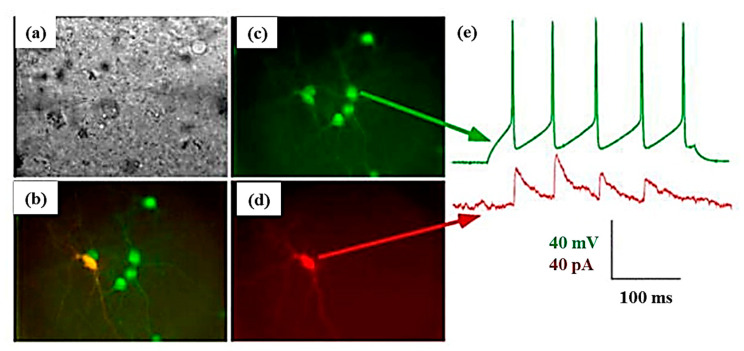
Testing of viral spread in the pre- and post-synaptic cells. (**a**) Image of the slice and recording pipettes with cells. (**b**) Merger of the fluorescent image. (**c**) Transfected cell with viral spread. (**d**) Transfected cell with TVA (cellular receptor for subgroup A avian leukosis viruses (ALV-A)) and rabies-virus glycoprotein (**e**) coinciding postsynaptic currents and action potentials in the cell with a monosynaptic connection. Reprinted with the permission of [107].

The rabies virus is a genetically modifiable virus that allows high-level expression of a specific gene in synaptically coupled neurons. The property is well suited for single-neuron analysis. A two-plasmid system has been utilized: one encoded replication-defective rabies virus RNA with the glycoprotein gene truncation and the other encoded only the glycoprotein. When electroporated into a single-neuron, the virus that assembled in one neuron lost its ability to replicate after it moved trans-synaptically (Figure 9). Analysis of the viral protein expression pattern would help to understand not only the pathogenesis of the rabies virus but also the neural connectivity in a dynamic fashion [107].

## 7. Single-Neuron Transfection Methods

The delivery of biomolecules into cells is an important strategy to investigate cell behaviors as well as the development of therapeutics. Conventional biological and chemical transfection agents, such as viral vectors [108], calcium phosphate, basic proteins [109], and cationic polymers [110], can deliver different biomolecules into cells and are suitable for general usages. However, most of these techniques are cell-type-specific bulk delivery and are often limited to low delivery efficiency and cell viability [111,112]. For example, certain viral vectors may be mutagenic to the transfected cells and can trigger immune responses and cytotoxicity [113]. Genes delivered via cationic polymers may be targeted to endolysosomes and result in endocytic degradation [114]. On the other hand, physical transfection methods use physical energy to create temporary pores on the cell membrane that allow foreign biomolecules into the cells by simple diffusion [115,116,117]. In the last two decades, due to the rapid development of micro- and nano-technologies, many physical techniques can deliver different sized biomolecules in different cell types (at a single-cell level) with high transfection efficiency and high cell viability [3,6,7]. The most commonly used physical transfection methods include microinjection [118,119,120], electroporation [121,122,123,124], optoporation [125,126,127,128], sonoporation, magnetoporation [129,130,131,132], and biolistic gene delivery [133,134,135]. The advantages and limitations of different single-neuron cell therapies and analyses are discussed below.

### 7.1. Microinjection

Microinjection is a versatile transfection method, suitable for almost all cells. The technique involves direct insertion of a hollow microneedle into a subcellular location of the membrane and delivers a precise amount of biomolecules into cells irrespective of their size, shape, and chemical nature [136]. The approach is quite labor-intensive and occasionally causes substantial stresses due to disruption of the plasma membrane, resulting in decreased survival rates of transfected neurons. Despite these drawbacks, microinjection has successfully delivered exogenous proteins, cDNA constructs, peptides, drugs, and particles into transfection-challenged individual neurons. One such example is the delivery of active recombinant enzymes (caspase-3, -6, -7, and -8) into individual primary neurons. The neurons displayed caspase-specific responses, including prolonged time-dependent apoptosis by caspase-6 (>0.5 pg/cell) [137]. The selectively toxic of Aβ_1–42_ via activation of the p53 and Bax proapoptotic pathway to only neurons was also proved by microinjecting Aβ_1–40_, Aβ_1–42_, and control reverse peptides Aβ_40–1_ and Aβ_42–1_ or cDNAs expressing cytosolic or secreted Aβ_1–40_ and Aβ_1–42_ in primary human neuron cultures, neuronal, and non-neuronal cell lines [138]. The mechanistic dissection of single-neural stem cell behavior in tissue was further evaluated by microinjection. The microinjection set-up consisted of a phase-contrast microscope with epifluorescence, trajectory, and micromanipulator [139]. Although current imaging techniques are equipped to monitor such behavior, the genetic manipulation tools are still devoid of achieving a balance between the gene expression and timescale for the singular gene product. Microinjection in mouse embryonic brain organotypic slice culture targeting individual neuroepithelial/radial glial cells (apical progenitors) avoided these shortcomings. The apical progenitor microinjection acutely manipulated the single-neural stem, and progenitor cells within the tissue and the cell cycle parameters otherwise indecipherable to apical progenitors in utero, go-through self-renewing divisions and neurons were produced. The microinjection of recombinant proteins, single genes, or complex RNA blends stimulated acute and distinct modifications in the behavior of apical progenitor cells and also changed the destiny of progeny [140]. Further, the role of two essential genes in mammalian neocortex expansion, namely the human-specific gene *ARHGAP11B* [141] and Insm1 [142] was assessed via microinjection.

**Figure 10:**
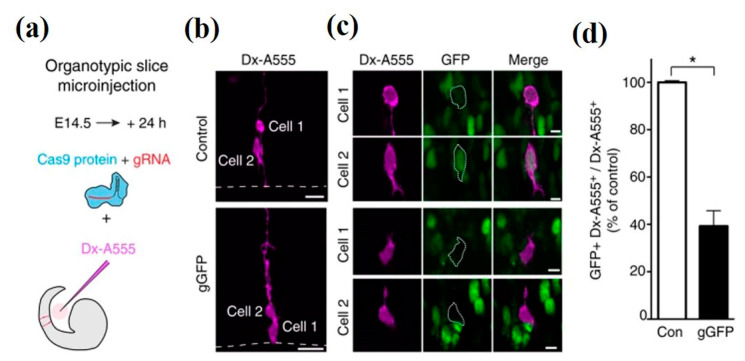
CRISPR/Cas9 (Clustered regularly interspaced short palindromic repeats- associated protein 9) -induced disruption of green fluorescence protein (GFP) expression in the daughter cells of single microinjected aRGCs in organotypic slices of the telencephalon of Tis21::GFP mouse embryos. (**a**) Scheme of the Cas9/gRNA complex microinjection. (**b**) Reconstruction of optical sections with maximum intensity projections for daughter cells of single aRGCs microinjected with either Cas9/control gRNA (top) or Cas9/gGFP (bottom) revealed by Dx-A555 immunofluorescence (magenta); cell 1, aRGC daughter; cell 2, BP daughter. Dashed lines depict ventricular surface. Scale bars, 20 μm. (**c**) Single optical sections of cells 1 and 2 shown in (**b**), showing the effects of Cas9 and control gRNA (top) or gGFP (bottom) on GFP expression. Scale bars, 5 μm. (**d**) Quantification of the proportion of daughter cells (Dx-A555^+^) of microinjected cells showing GFP expression 24 h after control (Con, white) or gGFP (black) microinjection. (* *p* < 0.05, Fisher’s test) Reprinted with the permission of [147].

Another study highlighted the fast and efficient CRISPR/Cas9 (Clustered regularly interspaced short palindromic repeats- associated protein 9) technology for the disruption of gene expression involved in neurodevelopment [143,144,145,146]. The technology eradicates the restrictions of transgenic knockouts and RNAi-mediated knockdowns. A radial glial cell (RGCs) in telencephalon slice of heterozygous E14.5 *Tis21*:: GFP mice were microinjected as shown in Figure 10a, to distinguish the progeny cells from the microinjected aRGCs. The microinjection cargo included recombinant Cas9 protein with either gRNA (gLacZ) or gGFP control. In this experiment, dextran 10,000-Alexa 555 (Dx-A555) acted as a fluorescent tracer for the aforementioned identification. Microinjection mainly aims single aRGCs in the G1 phase of the cell cycle, and therefore facilitates the monitoring of the CRISPR/Cas9-mediated disruption effect of gene (under observation) expression in the same cell cycle of the microinjected neural stem cell, as depicted in Figure 10b–d [147]. The microinjection mediated CRISPR technology provides new prospects for functional screenings and to determine the loss-of-function in the individual cell.

**Figure 11:**
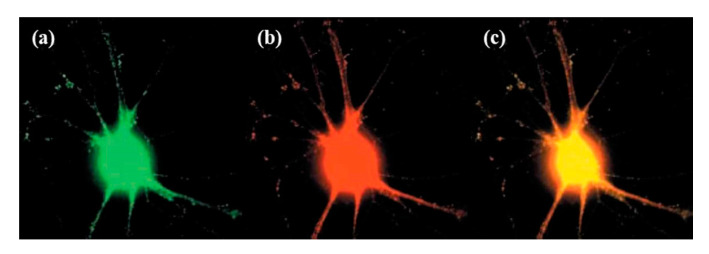
Cortical neurons expressing brain-derived neurotrophic factor (BDNF): (**a**) with green fluorescence protein after 24 h of delivery; (**b**) stained with anti-BDNF antibody; (**c**) merge image of both green fluorescence protein and anti-BDNF antibody. Reprinted with permission from [148].

Kohara et al. performed simultaneous injection of DNAs of green fluorescence protein tagged with brain-derived neurotrophic factor (BDNF) and red fluorescence protein (RFP) into a single-neuron (Figure 11). Thereafter, they visualized the expression, localization, and transport of BDNF in the injected single-neuron. This co-expression of two fluorescent proteins revealed the activity-dependent trans-neuronal delivery of BDNF [148]. Shull et al. recently developed a robotic platform for image-guided microinjection of desired volumes of biomolecules into single-cell. In this study, they delivered exogenous mRNA into apical progenitors of the neurons in the fetal human brain tissue. For the autoinjector, the injection pressure was set between 75 and 125 m bar, and it was microinjected from the ventricular surface to the depths of 10, 15, and 25 µm with the efficiency of 68%, 22%, and 11%, respectively. Thus, the autoinjector can deliver exogenous materials into targeted cells to the cluster of cells with high control and at single-cell resolution [119].

**Figure 12:**
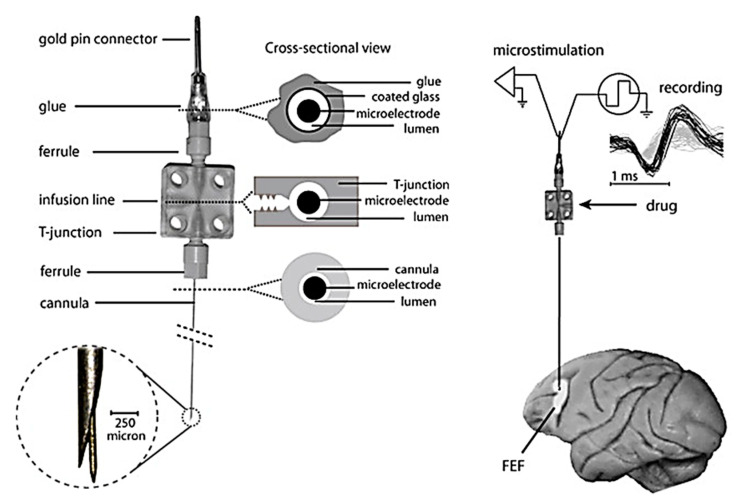
Microinjectrode system and its application. Briefly, a thin microelectrode passes through a 32 G cannula (OD: 236 m) which is connected to a T-junction via a ferrule. The electrode goes into a T-junction and a polyimide-coated glass tube with the terminal soldered to a gold pin. The polyimide tubing, gold pin, and ferrule are all pasted together. The middle part shows cross-sections through different parts of microinjectrode, i.e., the top ferrule, middle T-junction and bottom the cannula. An enlarged view of the microelectrode and cannula tips shows their relative position and size. A sample experiment is also displayed with single-neuron recording, electrical microstimulation and microinjection being performed in the frontal eye field (FEF). The single-neuron waveforms (black traces) segregated from background (gray traces) are also presented. Reprinted with the permission of [149].

A variant of microinjections has been formulated combining electrophysiology recordings, electrical micro-stimulation, and pharmacological alterations in local neural activity, most commonly used in monkey. The combination of the above-mentioned activities helps in providing a better way of explaining neural mechanisms [149]. Therefore, targeting simultaneous drug delivery, neurophysiological recording, and electrical microstimulation, various groups have developed “microinjectrode” systems. Sommer et al. established the primary connection between corollary discharge and visual processing via injectrode and segregating single cortical neurons. The results showed that spatial visual processing impairs if the corollary discharge from the thalamus is disturbed [150]. Crist et al. developed a microinjectrode which contains a recording electrode in addition to an injection cannula, facilitating simultaneous drug delivery and extracellular neural recording in monkeys. But the recording wire of the syringe typically recorded multi-unit activity, with frequent single-cell isolation [151]. Subsequently, modified injectrodes were introduced to achieve better recording quality and the ability to alter both neuronal activity and behavior in animals, an example being shown in Figure 12 with single-neuron recording, electrical microstimulation and microinjection in the frontal eye field (FEF), along with recorded single-neuron waveforms [84,149,152,153].

### 7.2. Electroporation

**Figure 13:**
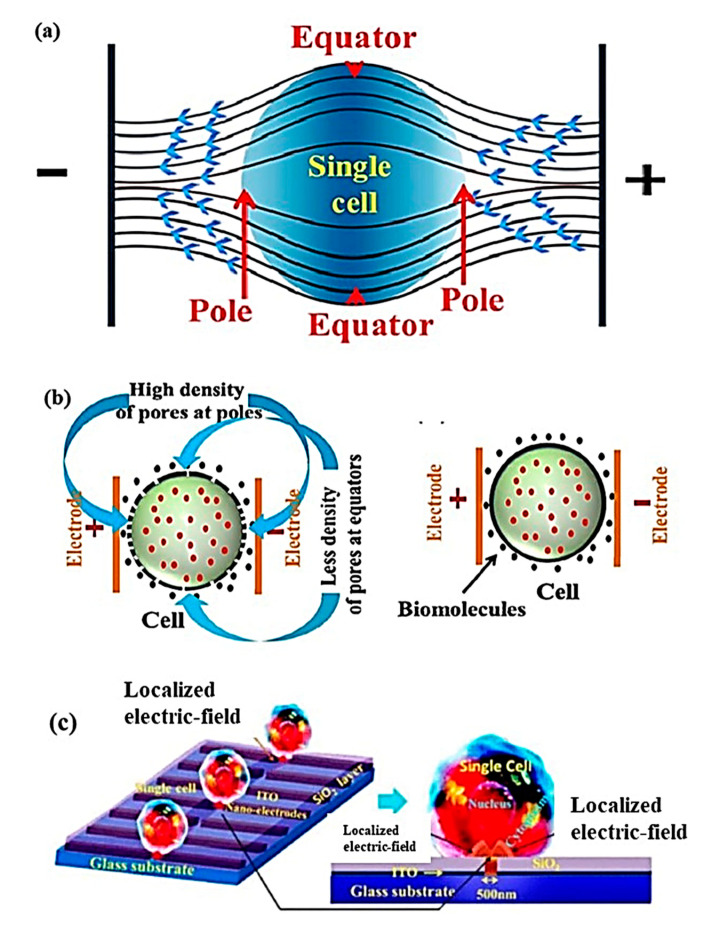
(**a**) Schematic showing distribution of electric field facilitating single-cell electroporation (SCEP); the induced transmembrane potential is found to be highest at the cell pole and decreases towards the equator. (**b**) Microfluidic SCEP with cell trapping. Reprinted with permission from [154]. (**c**) Localized SCEP with electric field (**b**) membrane area dependent density of pore formation and distribution due to non-uniform electric field application (**c**) nano-localized single-cell nano-electroporation. Reprinted with permission from [156].

Contrary to microneedles, single-cell electroporation displays better performance in specificity, dosage, cell viability, and transfection efficiency. Single-cell electroporation (SCEP) uses electric field application surrounding or a localized area of the single cell, with inter-electrode distance in the range of a micrometer to nanometer scale [154,155]. The application of a high external electric field in the vicinity of cell membranes increases their electrical conductivity and permeability owing to structural deformations occurring at the membrane for creating transient hydrophilic membrane pores and deliver biomolecules inside single-cell by simple diffusion process [156]. These transient pores are developed from the initial form of hydrophobic pores and therefore facilitate electroporation. The electric field can be applied in various ways, as shown in Figure 13: (a) non-uniform electric field distribution (higher field at poles and lower field at equators); (b) membrane area-dependent density of pores formation on single-cell due to non-uniform electric field application; and (c) nano-localized electric field application using nano-electrodes and biomolecular delivery [154,156].

The cell membrane surface subjected to electroporation is dependent on the nanochannel opening with diameter generally <500 nm and it could be constituted in the form of an array. The above-mentioned various types of set-up porate a small patch on the cell membrane, electrophoretically pushing polarized macromolecules inside the cell via the nanoscale pores [123]. Haas et al. originally used electroporation for studying the role of genes in the morphological development and electrophysiology of neurons in Xenopus laevis tadpole brain. They electroporated individual cells using electrical pulses from a DNA-filled micropipette. Single-cell electroporation was preferred due to the uniqueness of the individual neuron’s axonal and dendritic processes without any intervention from neighboring neurons’ processes. They also highlighted the role of gene expression on the transfected cell, and are either cell-autonomous or secondary because of interplay with transfected neighbors [123,157]. The most effective current for SCEP lies between 1 and 4 mA and the co-transfection rate for pGFP and pDsRed are greater with SCEP (96%), in comparison to whole-brain electroporation. Earlier dendritic growth of single-cell electroporated neurons in the tadpole brain can be examined only over six days [123]. Now it has been advanced to the level of the intact developing brain, where live two-photon fluorescence imaging shows the SCEP of a fluorescent dye or plasmid DNA into neurons within the intact brain of the albino Xenopus tadpole in the timescale of seconds to days without altering the neighboring tissues [158].

**Figure 14:**
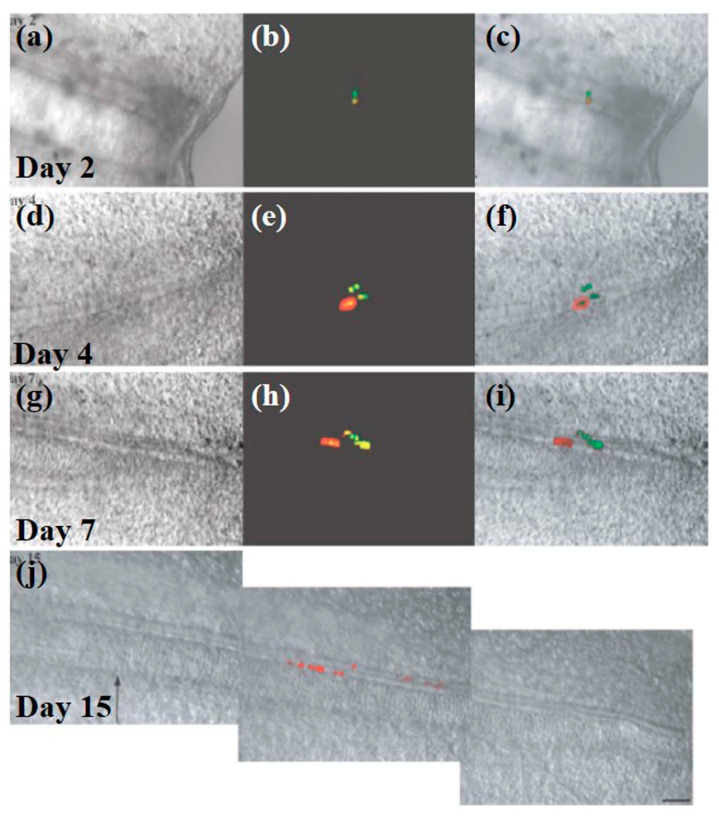
(**a**–**j**) Cell transfection is shown with cytoplasmic DsRed2-N1 and nuclear green fluorescent protein plasmids (**b**,**c**). The merged fluorescence and differential interference contrast (DIC) images after 2 days of amputation depict both the cells in the spinal cord with a distance of approximately 250–300 µm from the amputation plane (**c**). In the next 2 days, the cells undergo division and recruitment to the regenerating spinal cord (**e**,**f**). (The panels show only regenerating tissue.) The cell division continues and spinal cord growth continues rapidly (**g**–**j**). (**j**) A composite image of DIC images merged with the fluorescent image (15 days). Here, the initial two cells give rise to approximately ten cells on both the dorsal and ventral sides of the midportion of the developing spinal cord. The cell group is present over 560 µm length along the anterior/posterior axis. The original amputation plane is depicted by an arrow sign. Scale bar 100 µm in (**j**) (applicable to **a**–**j**). Reprinted with permission from [124].

Electroporation has been employed for the transfection of the spinal cord. The technique was initially amended for the transfection of single cells or small sets of cells inside the axolotl spinal cord, in the vicinity of the amputation plane. However, now it has attained advancements to allow the transfection of the labeled spinal cord cells, overcoming the requirement of transgenic knockouts or RNAi-mediated knockdowns [124]. Further, Echeverri and Tanaka tracked the explicit cell fate of neural progenitors present in the spinal cord via electroporation in tiny and transparent axolotls, transparent skin allows imaging of differentiating neurons with epifluorescence using differential interference contrast microscopy. As shown in Figure 14, the timeline of the growth of the regenerating spinal cord is as follows: progenitor cells recruitment from mature tissue to the regenerating part (day 2–4), cell-division (day 4–15), and cell-clones spreading along the A/P axis (day 7–15) [124].

Further in vitro electroporation and slice culture was performed for the interpretation of gene function in the mouse embryonic spinal cord owing to the low transfection efficiency of in utero spinal cord electroporation. The expression of the external gene in the embryonic spinal cord is governed by in utero electroporation. The axonal projections are unanimously directed from inside to the lateral side of the spinal cord. In comparison to neurons present in vivo, a single-neuron growing in the slice culture owns an extra number of complete neurites and therefore offers ease in the study of structural and behavioral alterations in individual neurons [159].

Electroporation has been shown to overcome the issues related to intracellular pressure resulting from injection or iontophoresis. Single-cell electroporation is simple, reproducible, highly efficient, and capable of introducing a variety of molecules, including ions, dyes, small molecular weight drugs, peptides, oligonucleotides, and genes up to at least 14 kb, into cells. The electrophysiological recording and anatomical identification by electroporation have been performed in a number of cells (CHO, HEK293, α-TN4 cells, etc), primary cultures of chicken lens epithelial cells [160] and retinal ganglion cells [161], using microelectrode and a few volts supplied from a simple voltage-clamp circuit. Graham et al. have demonstrated single-cell manipulations using a whole-cell patch type electrode, which can adapt to obtain electrophysiological responses easily using an amplifier that allows both a recording and stimulation mode [161]. Moreover, time-lapse in vivo electrical recordings of contralateral and ipsilateral, sensory-evoked spiking activity of individual L2/3 neurons from the somatosensory cortex of mice was also facilitated by using electroporation [162]. On-chip electroporation performed using micrometer-sized gMµE (an array of gold mushroom-shaped microelectrodes) device that enabled membrane repair dynamics and transient in-cell recordings [121]. Several additional devices with miniaturized and integrated microneedle electrodes or microchannels have been fabricated to perform single-cell electroporation [163]. These devices, consist of a wave generator, a biochip containing an array of microelectrodes, and a control system, permit the transfer of signals to a pre-selected single microelectrode of the biochip achieving the transfection of Cos-7 cells and single-neurons with oligonucleotides [164,165].

Further, optogenetic probes are also precisely targeted on individual neurons via single-cell electroporation. A targeted optogenetic expression among precisely grouped neurons helps in assessing the relation between neuron count, uniqueness, and spatial organization in circuit processing [165]. A similar approach will also help in the analysis of calyx-type neuro-neuronal synapses of the embryonic chick ciliary ganglion (CG) via single-axon tracing, electrophysiology, and optogenetic techniques. In vivo electroporation manipulated presynaptic gene and later 3D imaging was performed for single-axon tracing in isolated transparent CGs, followed by electrophysiology of the presynaptic terminal, and an all-optical approach using optogenetic molecular reagents [166] Long-term in vivo single-cell electroporation was conducted using Two Photon Laser Scanning Microscopy (2-PLSM) of synaptic proteins, combined with longitudinal imaging of synaptic structure and function in L2/3 neurons of the adult mouse neocortex. This result also expresses and longitudinally image SEP-GluR1 dynamics, suggesting a difference in spontaneous activity of synapses, and consequently, constitutive insertion through GluR1 receptors takes place [167].

**Figure 15:**
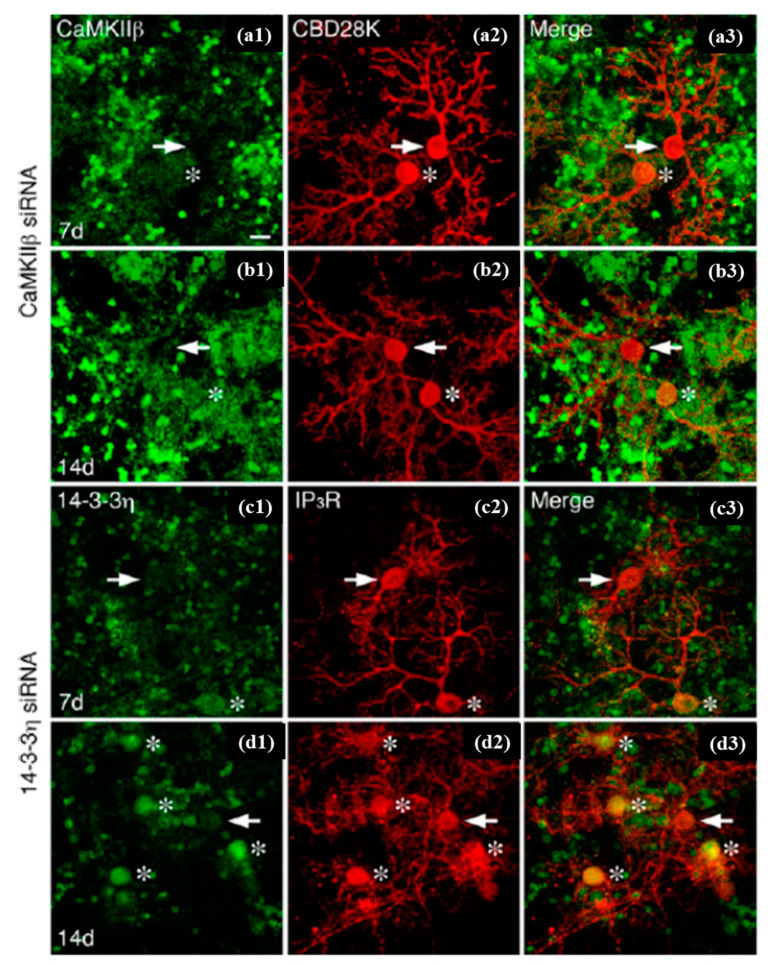
Immunostaining images of single-cell electroporated Purkinje cells small interfering RNA (siRNA) against calcium/calmodulin-dependent protein kinase ß (CaMKIIß) or 14-3-3η). SCEP was done at 11 days in vitro (DIV). The cell fixation was performed on day 7 (**a**,**c**) or day 14 (**b**,**d**) post electroporation (18 or 25 DIV, respectively) and double fluorescent immunostaining against CaMKIIß (green in **a**,**b**) and calbindin-D-28 K (CBD28K) (red in **a**,**b**) or 14-3-3η (green in **c**,**d**) and IP_3_R (red in **c**,**d**) was performed. Therefore, 1, 2 and 3 correspond to green, red and merged stains respectively. CaMKIIß or 14-3-3η signals decreased in electroporated Purkinje cells (arrows), but not in nearby non-electroporated Purkinje cells (asterisks). It is noteworthy that CaMKIIß and 14-3-3η expression was present for both Purkinje cells and granule cells. Scale: 20 µm. Reprinted with permission from [168].

Tanaka et al. performed single-cell electroporation and small interfering RNA (siRNA) delivery for gene silencing against the green fluorescent protein into GFP-expressing Golgi and Purkinje cells in cerebellar cell cultures. The temporal alterations in the GFP fluorescence (in the same electroporated cells) were observed for 4–14 days via repeated imaging (Figure 15). Furthermore, they checked the dependency of concentration for specific gene silencing and the non-specific off-target effects of siRNA inserted through this method, showing that the effects were present at least up to 14 days, yet differed between neuronal cell types [122,168].

Apart from the above-mentioned routes, single-neuron electroporation was performed on the cultured cortex to transfect gene encoding yellow fluorescence protein. Analysis of the dynamic of axon morphology indicated that electroporation had not affected developmental aspects [169]. Electroporation was also tested on an organotypic culture of hippocampal slices to introduce plasmids into single-neurons [170]. The approach has been used to demonstrate synthetic oligonucleotides delivery to identify duplex RNA and antisense oligonucleotide activators of human frataxin expression [171]. Using fluorescent Ca^2+^ indicator-loaded brain slices and in vivo samples, the morphology of the apical dendrites of several pyramidal neurons was found to be normal, indicating that the neurons had recovered from the electroporation procedure [172]. Single-cell electroporation accompanied by virus-borne genetically encoded Ca^2+^ sensors also allowed functionally trans-synaptic tracing in targeted single cells [173]. Single-cell electroporation was also used to identify and selectively label active homeodomain transcription factors mnx negative neurons in embryos of the double-transgenic line Tg(elavl3:GCaMP6f)Tg(mnx1:TagRFP-T) via two-photon confocal microscopy imaging [174].

### 7.3. Optical Transfection/Optogenetics

**Figure 16:**
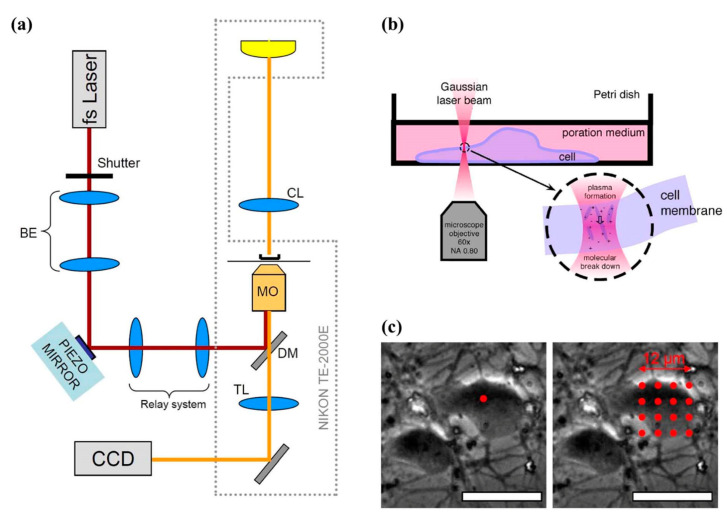
Optical transfection system using femtosecond laser (**a**) Schematic of the optical transfection system. (**b**) Side view of the Petri dish containing a single-neuron for transfection. (**c**) Irradiation patterns (red dots) superimposed on phase-contrast images of cortical neurons. Reprinted with permission from [175].

Antkowiak et al. designed a technique with an image-guided, three-dimensional laser-beam steering system for transfecting specified cells (Figure 16). Channelrhodopsin-2 (ChR2) was successfully introduced into a large number of cells in a neural circuit individually in a sequential manner as shown in Figure 16b,c. This technique enabled the transfection of selective cells on a large-scale basis and performed rapid genetic programming of neural circuits [175]. Barrett et al. successfully phototransfected primary rat hippocampal neuron with a Ti-sapphire p laser using 100 fs pulses with 30 mJ power, and 1–5 ms pulse duration. Successful transfection of the neuron could be observed after 30 min of laser exposure [176].

Optogenetics are now widely used for activation and silencing of neuron populations defined by their molecular and activity profiles and projection patterns [177]. Some commonly used tools are light-gated ion channels (e.g., channelrhodopsin-2, or ChR2) and ion pumps (halo-rhodopsin or archaerhodopsin-3). These molecules, combined with a suitable optical method, can trigger their function to control neuronal activities. Owing to low channel conductance of ChR2, single-cell optical stimulation has not been feasible previously [178]. Simultaneous activation of a large number of channels can help to achieve sufficient depolarization up to a space of tens of µm^2^. Nevertheless, conventional one-photon and two-photon scanning imaging systems addressing this issue inevitably activate neurons in an untargeted fashion. Though these studies showed high spatial resolution, yet the required activation time for large area appropriate for firing action potentials was approximately 30 ms. The two-photon temporal focusing (TEFO) technique developed earlier in this decade may realize the demand. The system has an independent axial beam profile from lateral distribution and simultaneous excitation of multiple channels on individual neurons, resulting in strong (up to 15 mV) and fast (≤1 ms) depolarizations. The techniques may allow quasi-synchronous activation of neurons along with specific cellular compartments. The TEFO with a conventional dual galvanometer-based scanning system repositions the excitation spot in a rapid manner typically <0.2 ms to any point in a 100 μm field. The precise spatial and temporal control of firing activity performed with a single or preferred several single cells, particularly while combining with selective ChR2 expression of specific population of cells. This technique highlights the scope for detailed, high-throughput analysis of connections and neural network dynamics and evaluation of the functional significances of their activation both in vitro and in vivo [179].

**Figure 17:**
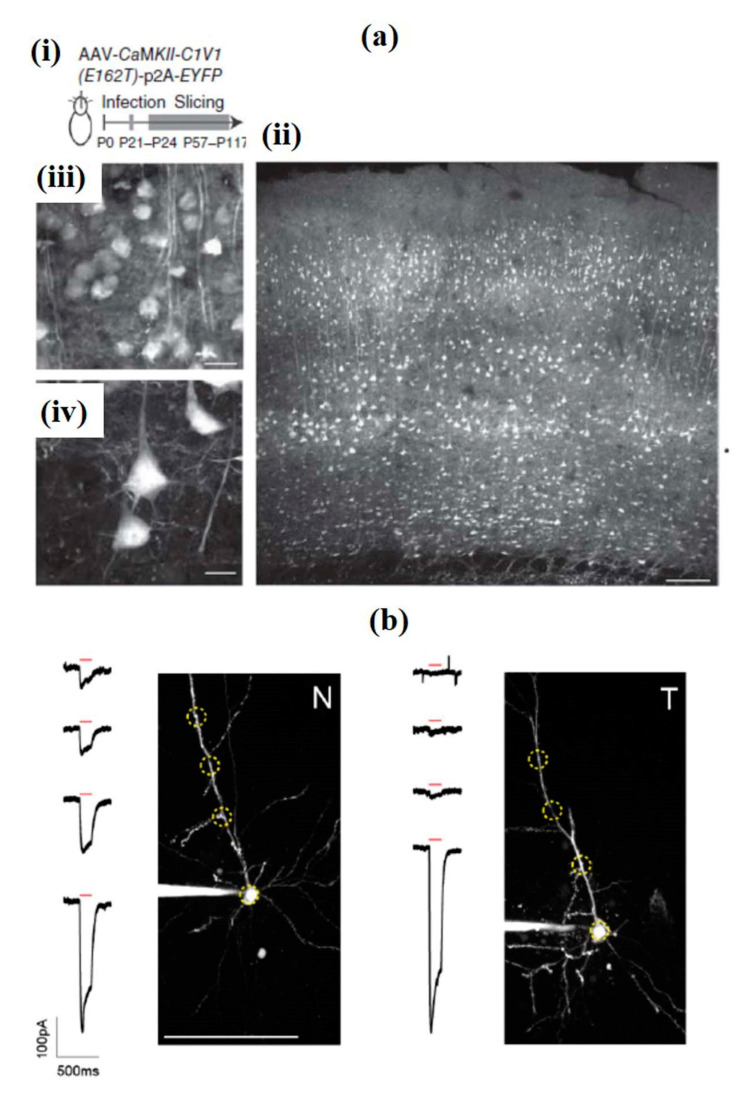
(**a**) Two-photon activates of individual neurons present in mouse brain slices with C1V1T. (**i**) The experimental scheme shows the opsin *C1V1T* and *EYFP* genes encoded by Adeno-associated virus (AAV) are inserted in the somatosensory cortex of the mouse. Brain slices were prepared at a designated time point from the infected region. (**ii**) Two-photon fluorescence image of a living cortical brain slice expressing EYFP (940-nm excitation, 15 mW on the sample, 25×/1.05-NA objective; scale bar, 100 μm). (**iii**, **iv**) Magnified images from (**b**) show cells with C1V1T-expression present in higher (**iii**) and lower (**iv**) layers (scale bars, 20 μm (**iii**), and 10 μm (**iv**) Reprinted with permission from [180]. (**b**) Illustrative two-photon highest intensity projections of Alexa 594 fluorescence and current responses against a single 150 ms temporal focusing (TF) stimulation pulse (red bar) for patched and dye-filled pyramidal cells present in acute slices expressing targeted (T) and nontargeted (N) ChR2. Scale bar = 100 mm. Reprinted with permission from [181].

To overcome the limitation of the requirement of high opsin expression and complex stimulation techniques, Packer et al. used a new red-shifted chimeric opsin C1V1_T_ formed by combining ChR1 and VChR1 (Figure 17a). This technique involved a spatial light modulator, in which the laser beam was split and targeted to several positions in a neuron, allowing simultaneous optogenetic activation of selected neurons in three dimensions. The method also showed the possibility to optically map short-term synaptic plasticity. Figure 17b shows the effect of a single 150 ms TF stimulation pulse (red bar) via two-photon highest intensity projections of Alexa 594 in the form of fluorescence and current responses for patched and dye-filled pyramidal cells in acute slices expressing targeted (T) and nontargeted (N) ChR2 [180].

To avoid undesired neuron labeling and studies, a combined temporal focusing with the spatial confinement of ChR2 expression to the neuronal cell body and proximal dendrites were also tested. This was based on the Kv2.1 potassium channel, which has a particularly unique localization to clusters at the neuronal soma and proximal dendrites. As shown in Figure 17b, the action potential was evoked in individual neurons, and peak generation took place with GCaMP6s, and functional synaptic connections with patch-clamp electrophysiological recording could be determined at a single-neuron resolution [181]. Another study also presented a conventional optogenetic two-photon mapping method in mouse neocortical slices by activating pyramidal cells with the red-shifted opsin C1V1, while recording postsynaptic responses in whole-cell configuration. The use of temporal-focused excitation or holographic stimulation, as in earlier method, limits the problem of dendritic activation, yet the current method is simple and fast [182].

Contrary to the above-mentioned single-cell resolution optogenetics, sometimes neurons own high expressing opsins so that even two-photon (2P) stimulation of a single-neuron soma is sufficient to excite opsins present on crossing dendrites or axons along with stray excitation of neighboring neurons. Therefore, the localization of a novel short amino-terminal peptide segment of the kainate receptor KA2 subunit 18 fused with high-photocurrent channelrhodopsin CoChR19 in neuron soma avoided crosstalk and facilitated selected handling of CoChR to neuron soma in mammalian cortex. The combined holographic 2P stimulation using low-repetition fiber laser optogenetically stimulated single cells present in mammal brain slices. The use of light pulses with subtle powers lead to zero-spike crosstalk with neighboring cells and a shown temporal resolution of <1 ms. It also implemented protein fusion known as somatic CoChR (soCoChR), along with parametrized 2P stimulation enabled probing of various circuit neural codes and computations. The 2P computer-mediated holography sculpts light for simultaneously lighting many neurons in a network while maintaining the standard temporal precision to precisely stimulate neural codes [183]. The expression of some opsins is restricted genetically within the somatic part of the neurons; it offers a crucial feature of eliminating spurious activation of nontargeted cells while causing excitation of multiple neurons. Also, parallel illumination of conventional ChR2 and slow opsins such as C1V1 and ReaChR have fired up to 20–30 Hz spike with susceptibility to spike duration changes and the generation of spurious extra spikes. The problems are due to the limited kinetics of opsins. Certainly, high-frequency, light-driven action potential (AP) trains need opsins with rapid off kinetics maintaining fast membrane repolarization and inactivation recovery after every spike. All these facts postulate one hypothesis, that the in-depth optical regulation of neuronal firing with high spatiotemporal precision is dependent on 2P parallel photostimulation of fast opsins. Therefore, 2P action spectrum and kinetics of the fast opsin Chronos with holographically shaped light pulses were characterized. It was demonstrated that efficient current integration with 2P parallel illumination, enabled AP generation with sub-millisecond temporal precision and neuronal spike frequencies up to 100 Hz. The use of a fiber amplifier and high-energy pulse laser decreased the average illumination power many-folds. The outcome suggested mimicry of a broad range of physiological firing patterns with sub-millisecond temporal precision, as it is critical for understanding the relationship between behavior and pathological states in terms of particular patterns of network activity [184].

Another research article computationally predicts the power of external regulation of the firing times of a cortical neuron following the Izhikevich neuron model. The Izhikevich neuron model helps to follow the membrane potential values and firing times of cortical neurons efficiently and in a biologically possible way. The outside regulation is a simple optogenetic model including an illumination source, which stimulates a saturating and decaying membrane current. Here, the firing frequencies are assumed to be significantly lower for the membrane potential to achieve resting potential after firing. The model fits neuron charging and recovery time along with peak input current, to derive lower bounds on the firing frequency, achievable without significant distortion [185].

## 8. Micro/Nanofluidic Devices for Single-Neuron Analysis

In the last two decades, the rapid development of micro/nanotechnologies and their integration with chemistry, chemical engineering, and life science have encouraged the emergence of lab-on-a-chip devices or micro-total analysis systems (μ-TAS), which are powerful tools used to perform a variety of cellular analyses. The devices are capable of performing precise single-cell and subcellular analyses with minimal sample consumption. Micro/nanofluidic devices can create optimal microenvironments for growing cells and guiding their growth direction, especially for neurons. Microenvironments within micro/nanofluidic devices can enhance the axonal growth and can dissolve molecules and can create contact-mediated signaling from guided cells and cellular matrix [186].

The neurochip with microwells and microchannels along with planar multielectrode arrays to confine single-neuronal cells were designed and used to study the cell electrophysiological activity. A PDMS film with varying microwell sizes for cell patterning was fabricated on glass substrates with 40 µm wide ITO electrodes. The cell patterning structures restricted the movement of soma by allowing only the neurites to extend through the microchannel. Thus, one-to-one neuron electrode interfacing was established along with patterned structures and planar multielectrode arrays [187].

**Figure 18:**
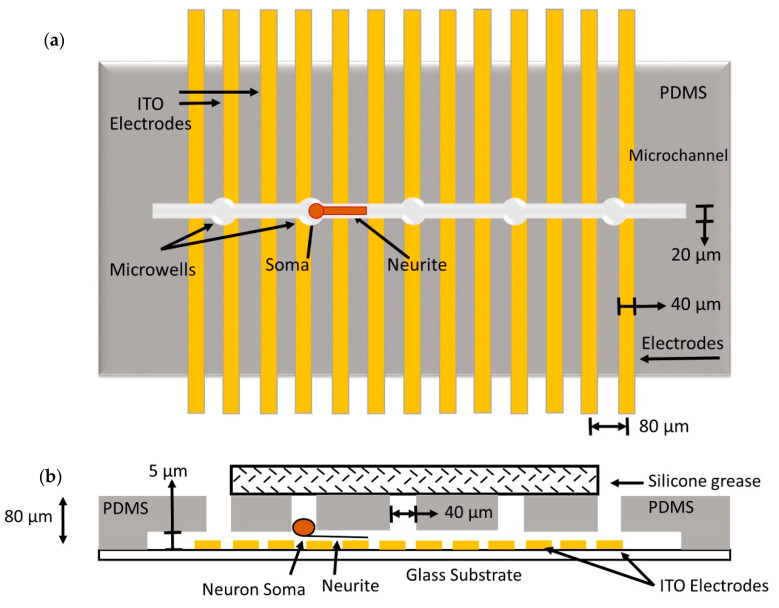
Schematic showing a microelectrode device fabricated by photolithography with microwells and microchannels on a planar multielectrode array, in which neurons were individually positioned in microwells, view from top (**a**) and side (**b**). Redrawn from [188].

This study was further extended by integrating a substrate with a multielectrode array for recordings purpose from extended neurites in individual microchannels, as shown in Figure 18. The activity of extended neurite from the microwell was recorded by 18 electrodes, and a density analysis of single-cell current was carried out. By using this technique, the electrical stability of the electrode-neuron interface was enhanced, in comparison with the other using a planar multielectrode array [188].

A biochip with asymmetrical channels was developed to study the polarized axonal growth in neural circuitry. This device consisted of microwells connected by numerous micro tunnels, which served as a guidance for developing axons to reach target neurons. A laser-based cell deposition system was used to place single cells into specific microwells in the device. The design of asymmetric channels improved the polarity as well as connectivity of the individual neurons [189]. Another asymmetric microchannel platform consisting of independent cell culture chambers, separated by axonal diodes, which helped to achieve required directionality for growing single-neurons. The neuronal cells were cultured in a way that the cell somas were retained in the microchamber, while the axon of a single-neuron extended to the other chamber through the axon diode. The axon diode had a decreasing cross-section from the culture chamber of 15 µm to the target chamber of 3 µm, hence enhancing the directionality and synapse formation. This device helped to study neuronal development and synaptic transmission and hence it can be developed further to study neurodegenerative diseases such as Alzheimer’s, Parkinson, and Huntington diseases [190]. A similar type of device including symmetric but smaller microfluidic channels also showed unidirectional extension of axons. By using this device, degeneration and regeneration of individual axons were studied by injuring the extended axons along the microchannels with the help of femtosecond laser. It was noticed that even after the injury, the axons tend to extend to the target chamber, and hence this device enabled a better understanding of neuronal response to injury [191].

**Figure 19:**
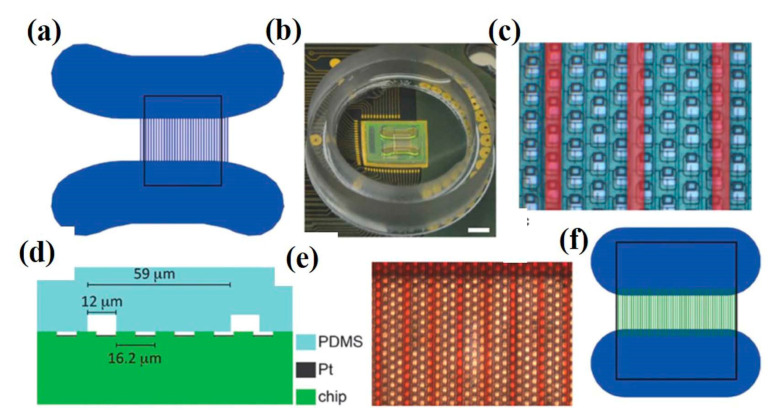
(**a**) The geometry of the microfluidic device on the microelectrode array. (**b**) Image of the packaged chip with the device on the top. (**c**) Magnified image of the electrodes and the channels; channels are highlighted with red, and the scale bar is 10 µm. (**d**) Cross-section of chip depicted with dimensions. (**e**) The images of the channels are highlighted in red; the scale bar is 20 µm. (**f**) The device with a small chamber and channels with an array marked inside the black box. Reprinted with permission from [193].

A silicon-based device with a patch-clamp microchannel array that acts as a cell-trapping platform has been designed for the electrical recording of single-neurons. The device consisted of two fluidic compartments with a cell injection chamber at the top layer and six independent microchannels and microholes at the bottom compartment. The local perfusion of single-neurons was obtained by controlling pressure in the microfluidic compartments. The device had a successful trapping rate of approximately 58%, which facilitated further analysis of the trapped cells in electrical recording and drug screening applications [192]. Figure 19 shows a microfluidic device with a complementary metal–oxide–semiconductor microelectrode array, which was designed to study the axonal signal behavior of single-neurons. This device consisted of two neuronal culture chambers connected by 30 microchannels with 12 µm width and about 10–50 microelectrodes were fabricated along each channel. This study revealed that the electrical activity of soma could be related to its axons, and the single action potential propagating along the long length of individual axons with high spatial resolution can be recorded [193].

The first neurochip was a silicon-based micromachined device with a 4 × 4 array of metal electrodes, which allowed growth and monitor neuronal cell individually. In the neurochip, neurowells were designed to capture the soma, while the neurites extend to gold electrodes, which was fabricated on the bottom of the chip. This device was designed to mechanically trap a neuron near an extracellular electrode of the multielectrode array with electrodes surrounded by micro tunnels. When an individual neuron was trapped onto an electrode site, the cell soma was captured inside it, allowing only the neurites to propagate along micro tunnels. The biochip yielded high neuronal cell viability and the action potential of each neuron was detected by each electrode, and there was no crosswalk between the channels [194]. These micro tunnels help the neurites from different neurons to form neural connections and can be recorded to study synaptic connections [195].

**Figure 20:**
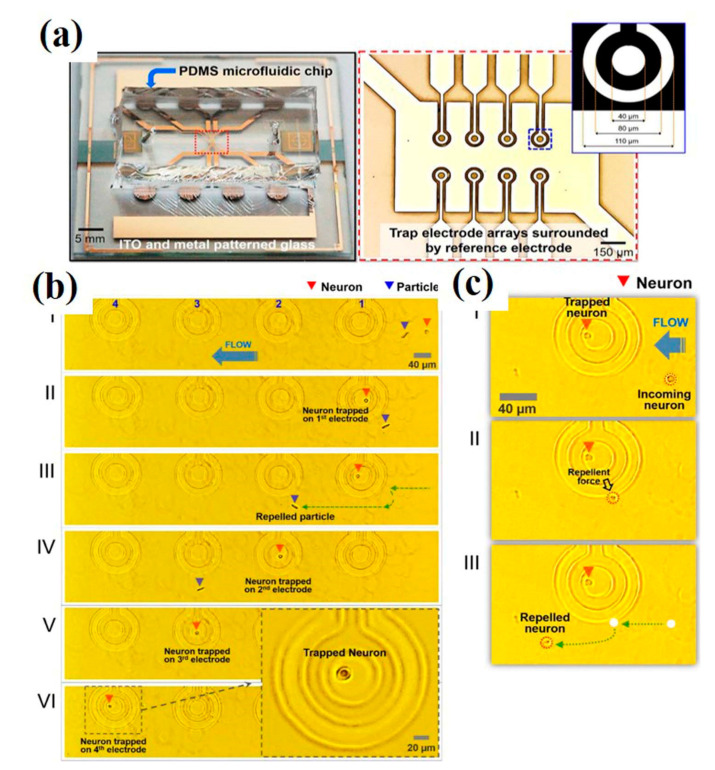
(**a**) Image of the microfabricated device and bright-field microscopic image of the electrode array. (**b**) Recorded images of single-neuronal cell manipulation on the array of ring-shaped traps. Incoming neuron (**I**) entering the 1st trap. (**II**) The neuron gets immobilized in the 1st trap electrode against a fluid flow. (**III**) When a neuron is trapped, the repelled particle keeps on moving in the flow of media. (**IV**) The released neuron gets trapped in the 2nd trap. (**V** and **VI**) The neuron is trapped in the 3rd and the 4th ring trap in turn. (**c**) The images show bouncing motion of the neuron subjected to a repulsive force. When the target neuron gets trapped in the desired electrode, the incoming neuron faces repulsion due to DEP force. At the end, when the incoming neuron reaches the outside of the electrode, the repulsive force pushed the neuron out of the ring. Reprinted with permission from [58].

A compartmentalized microfluidic device integrated with microelectrode array was designed to study activity-dependent dynamics in single-neurons and synaptic networks. The device has three microfluidic chambers, presynaptic, synaptic, and postsynaptic chambers (each) with axonal, reference, and postsynaptic electrodes to record the activity of single projecting axon. These presynaptic axons were recorded selectively by placing electrodes under the presynaptic chamber, and this study was further extended to study calcium dynamics [196]. Figure 20 depicts a microfluidic DEP device consisted of a PDMS microfluidic chip with ring-shaped indium tin oxide microelectrode array. In this device, the single-neuron was selectively trapped into the electrode, and the other neurons in the vicinity of the electrode were repelled by the DEP force. The amplitude and frequency of alternating current used to trap cells on the electrodes were 8 Vpp and 10 MHz, respectively. The trapped neuron was recorded, and its morphological changes were tracked with the assist of a phase-contrast microscope. Thus, this device enabled us to study multiple single-neurons at the same time and also electrical communication between them [58].

## 9. Artificial Intelligence and Single-Neuron

With advances in technology and instrumentation sensitivity, huge data is generated, but variation between batches in inevitable with enhanced susceptibility. In spite of the application of several correction models, the result is dependent on the actual magnitude of the effect [197]. Therefore, artificial intelligence is being employed to stimulate the learning processes otherwise occurring in humans, i.e., neural networks. Accelerated brain research initiatives are relying on AI-based tools, despite the different approaches, emphases and routes of neural studies. In spite of different research domains in the field of neuroscience employing different approaches and methodology, all have same objective of developing the next generation of AI-based tools [198,199]. For example, the Brain Research through Advancing Innovative Neurotechnologies (BRAIN) Initiative is moving forward to bring revolution in machine learning through neuroscience. As per the scope of this review paper, single-neuron analysis comes with several challenges, i.e., the curse of dimensionality, sparsity, degree of noise, batch errors, and data heterogeneity, which often hinder the performance of conventional computational approaches to scale up as data complexity and size grow, making the platform for contemporary deep learning algorithms. Processing and interpreting such high-dimensional single-cell information increasingly challenges conventional computational informatics calling for powerful and scalable deep learning models for dropout imputation, cell-subtype clustering, phenotype classification, visualization, and multi-omics integration. Iqbal et al. developed a fully automated AI-based method for whole-brain image processing to Detect Neurons in different brain Regions during Development (DeNeRD—Detection of Neurons for Brain-wide analysis with Deep Learning). This method to detect neurons labeled with various genetic markers is based on the state-of-the-art in object detection networks called the Faster Regions with Convolutional Neural Network (Faster RCNN) [200]. Further, a deep learning platform was developed for the identification and segmentation of active neurons. The core component consists of 3D CNN named STNeuroNet.re derived employing the two-sided Wilcoxon rank sum test. STNeuroNet was conceptualized on the basis of DenseVNet, a deep learning platform consisting of 3D convolutional layers, for the segmentation of active neurons from two-photon calcium imaging data. The STNeuroNet is equipped to extract relevant spatiotemporal features from the imaging data without prior modelling [201]. The next area in which deep neural networks have been employed for single-neuron analysis is single-cell RNA sequencing (scRNA-seq) data. An accurate, fast and scalable DeepImpute “Deep neural network Imputation” imputes single-cell RNA-seq data; outperforming the efficiency of other methods like mean squared error or Pearson’s correlation coefficient as the dataset size increases [202]. During scRNA-seq, sometimes noise due to amplification and dropout may obstruct analyses, therefore the need for scalable denoising methods arise. A quality, high speed deep count autoencoder network (DCA) was proposed to denoise scRNA-seq datasets. This takes the count distribution, overdispersion and sparsity of the data into account using a negative binomial noise model with or without zero-inflation, and nonlinear gene-gene dependencies are captured. It is possible to work with datasets from millions of cells owing to the linear scaling with the number of cells [203]. Another single cell-based model scDeepCluster was developed to overcome the statistical and computational challenge during the Clustering transcriptomes profiled by scRNA-seq to reveal cell heterogeneity and diversity [204]. Therefore, lately, deep learning is an ideal choice for big data integration or testing. However, a major concern around deep learning methods is the “black-box” nature of the models and their un-interpretability due to the huge number of parameters and the complex approach for extracting and combining features. While the data science community is active in enhancing interpretability of deep neural networks, further research in biomedical contexts is required to understand clinically or biologically relevant patterns in data raised to accurate predictions, and to improve the users’ trust ensuring that the model decides based on reliable reasons rather than artifacts in data.

## 10. Limitations and Future Prospects

The current review includes the merits and limitations of single-neuron analysis. As discussed, the single-neuron-at-a-time methodology amalgamated with complementing technologies allowing recordings or imaging groups of neurons helps build a better understanding of complex neural networks. A recently developed, sophisticated electrophysiology and connectivity tool, named Patch-seq, associated with neuronal activity visualization and manipulation platform can assist in outlining the connections and functions of each neuronal type. Similarly, another technique, named scRNA-seq, elucidates the cell types in the brain via single-cell sequencing methods, single-cell genomics, epigenomics (including methylation, mapping, sequencing, DNA accessibility, and chromosome conformation), and multi-omics. These tools help in the decoding development stages, epigenetics, and functionality of the brain at single-cell resolution. But sometimes, the connecting RNA techniques require in few micrograms, corresponding to several cells, presenting the scope in this front. Moreover, the difference in spatial positions, temporal points, and poor health stages may cause variation in the analysis.

In recent years, advanced techniques facilitate automatic and high-throughput single-cell trapping followed by sequencing along with analyzing large datasets. All of these techniques motivate and strengthen the upcoming research activities in the direction of preparing an all-inclusive human brain cell atlas. But the rate of data production raises a challenge to process and make sense of it. Based on the processing of data, many scientists can make discoveries daily by employing new computational methods. On the other hand, droplet-based sequencing can produce scRNA-seq datasets covering >5 × 10^5^ single cells, comprehends speed, and memory adeptness to state-of-the-art tools.

As stated earlier, multiple studies reported differences in cell types, number, cell cycle stage, extracellular matrix, and cell networking in different parts of the brain. Herewith, efforts are needed to integrate cell types from various studies. Hence, the biggest problem occurs on the level of scale in different reports. Additionally, from the aspect of multiplexity, current multiplexing is still not enough for whole proteomics detections (>10,000 proteins in a single cell). During standard bulk analysis, data reproducibility can be controlled owing to multiple biological and technical replicates. Single-cell experiments, particularly for scRNA-seq, contain the inability to replicate measurements on the same cell, and single-cell data is generally full of noise owing to technical variations occurring in multiple-step processes. Also, the biological variations arise as a result of cellular level heterogeneity, therefore increasing the sensitivity of scRNA-seq workflow at multiple levels, ranging from sample preparation, library preparation and sequencing and data analysis towards technical inconsistency and batch effects.

Apart from biological differences, experiment methodology, processing, handling as well as data processing workflows make it difficult to get a comparable result from the same model of the diseased or normal brain at any scale, i.e., organ, tissue, or at the level of an individual neuron. Therefore, the experimental protocols and computational outlines based on including and comparing scRNA-seq data from various platforms would overcome this issue. Lately, linked inference of genomic experimental relationships (LIGER) has proven useful in integrating multi-omics single-cell sequencing data [205]. Finally, single-cell multi-omics is going to gain huge success for brain studies by integrating data from various platforms. The classification of retinal bipolar cells has been the best-suited example of this set-up. [206]. The classification took cues from different techniques as well, i.e., structure and morphology (electron microscopy), electro-physiology (calcium imaging), and molecular biology (scRNA-seq) data. For better consideration of network organization and functioning of the brain, there is a great need for the unprejudiced, methodical assembly of molecular, morphological, physiological, functional, and connectivity data.

Overall, the knowledge of the brain is still in its infancy, but the rapidly growing single-cell sequencing technologies have already gathered ample data for future assessment and presented a never before seen map of the brain with single-cell resolution. Therefore, despite a range of complications and challenges, overwhelming progress is anticipated in the upcoming decade.

## 11. Conclusions

This review provides a broad perspective to the readers about the recent advances in single-neuron activity, neural circuit designing, and their sensitivity. We also emphasize in detail the current progress and future trends of single-neuron behavioral analysis, including the models, isolation, mapping, and electrophysiological recording. So far, isolation of single-neurons and maintaining their viability is still a challenging task. The advanced imaging and manipulating tools would continue to decipher the rise of thoughts and actions in the human brain. The details of single-neuron manipulation, isolation, sequencing, transfection, and analysis were elaborated using recent developed micro/nanofluidic devices as well as some physical methods, such as microinjection, electroporation, and optogenetics. The single-neuron optogenetics reveal the fundamental information about the sparseness of representations in neural circuits. Mapping neural connection at single-cell resolution would encourage planning systematic physiological experiments, probing connectivity between hundreds or thousands of neurons. Alongside this, deep learning is a promisingly potent machine learning technology, and the ongoing research in this field is expected to reign over the recent “big bang” of single-neuron data, just like it has been doing in other fields. The amalgamation of sophisticated visualization hardware, software, and huge neuro-anatomy data has supported the interpretation of decades of cumulative knowledge into a human axonal pathway atlas, which would be key for educational, scientific, or clinical investigations in future. However, we have made remarkable achievements in the field of human neuroscience, always accompanied by real-world problems.

## Figures and Tables

**Table 1:** Single-neuron models, including the year of model proposal, model category, type of model, keynotes, and drawbacks.

Model Name	Proposed Year	Category of Model	Type of Model	Keynotes	Drawbacks
McCulloch and Pitts	1943	Computational model.	Simplified neural models.	-	Problems of sense awareness, perception, and execution [21].
Hodgkin and Huxley model	1952	Electrical input–output membrane voltage models.	Neuronal membrane model with voltage-sensitive potassium and sodium channel.	Mechanism of generation and propagation of action potential. Fourth-order system of the nonlinear ordinary differential equation.	The generation of electric signals and action potential propagation is not explained [22,23].
Multi-compartmental model	1991	Electrical input–output membrane voltage models.	Biophysical neuron model.	Four compartments in series (one dendrite dividing into three series-coupled segment D).	It depends on only one parameter [24].
FitzHugh–Nagumo model	1961–62	Electrical input–output membrane voltage models.	2D simplified model of Hodgkin and Huxley model. Qualitative model	Neuronal excitability and spike-generating mechanism.	Unrealistic model to elucidate the mechanism of some function of the neuron [25,26,27].
Hindmarsh–Rose model	1984	Electrical input–output membrane voltage models.	A generalized model of the FitzHugh-Nagumo model. Classic model used to study bursting behavior.	A fast subsystem to generate action potentials. A slow subsystem to modulate spiking pattern. Slow ion channel to elucidate the mechanism of isolated burst and periodic bursting.	The physical meaning of variables x, y, z was not explained [28,29].
Integrate and Fire model	1907	Electrical input–output membrane voltage models.	Spike generation.	Excitability of neuron, Neural coding.	No time-dependent memory [18,30].
Leaky Integrate and Fire model		Electrical input–output membrane voltage models.	Spike generation.	Excitability of neuron.	Unrealistic behavior and inaccurate frequency response of real neurons [31].
Hopfield model	1982	Electrical input–output membrane voltage models.	Associative memory network.	Mechanism of distributive memory.	The plasticity of the synapse was not discussed [16,32].
